# A comparison of the molecular organization of genomic regions associated with resistance to common bacterial blight in two *Phaseolus vulgaris* genotypes

**DOI:** 10.3389/fpls.2013.00318

**Published:** 2013-08-29

**Authors:** Gregory Perry, Claudia DiNatale, Weilong Xie, Alireza Navabi, Yarmilla Reinprecht, William Crosby, Kangfu Yu, Chun Shi, K. Peter Pauls

**Affiliations:** ^1^Department of Plant Agriculture, University of Guelph, GuelphON, Canada; ^2^Department of Biological Sciences, University of Windsor, WindsorON, Canada; ^3^Agriculture and Agri-Food Canada, c/o Department of Plant Agriculture, University of Guelph, GuelphON, Canada; ^4^Greenhouse and Processing Crops Research Centre, Agriculture and Agri-Food Canada, HarrowON, Canada

**Keywords:** common bacterial blight, next generation sequencing, disease resistance, comparative genomics, phaseolus vulgaris

## Abstract

Resistance to common bacterial blight, *caused by Xanthomonas axonopodis* pv. phaseoli, in *Phaseolus vulgaris* is conditioned by several loci on different chromosomes. Previous studies with OAC-Rex, a CBB-resistant, white bean variety of Mesoamerican origin, identified two resistance loci associated with the molecular markers *Pv*-CTT001 and SU91, on chromosome 4 and 8, respectively. Resistance to CBB is assumed to be derived from an interspecific cross with *Phaseolus acutifolius* in the pedigree of OAC-Rex. Our current whole genome sequencing effort with OAC-Rex provided the opportunity to compare its genome in the regions associated with CBB resistance with the v1.0 release of the *P. vulgaris* line G19833, which is a large seeded bean of Andean origin, and (assumed to be) CBB susceptible. In addition, the genomic regions containing SAP6, a marker associated with *P. vulgaris*-derived CBB-resistance on chromosome 10, were compared. These analyses indicated that gene content was highly conserved between G19833 and OAC-Rex across the regions examined (>80%). However, fifty-nine genes unique to OAC Rex were identified, with resistance gene homologues making up the largest category (10 genes identified). Two unique genes in OAC-Rex located within the SU91 resistance QTL have homology to *P. acutifolius* ESTs and may be potential sources of CBB resistance. As the genomic sequence assembly of OAC-Rex is completed, we expect that further comparisons between it and the G19833 genome will lead to a greater understanding of CBB resistance in bean.

## Introduction

*Phaseolus vulgaris* L. (common dry bean), a member of the Fabacaea family, is the most important food legume in the world and is widely cultivated in Asia, North and South America and Africa, The *Phaseolus* genus is comprised of over 50 members, with *P. vulgaris* being the most commonly cultivated species. The primary gene pool of *P. vulgaris* is comprised of two geographically isolated pools: the Mesoamerican, and the Andean (Evans, 1973; Koening and Gepts, 1989; Koening et al., 1990; Singh et al., 1991; Debouck et al., 1993), which are distinguished by differences in seed size and color that define different market classes, although, in general, the beans of Mesoamerican origin have smaller seeds than those of Andean origin (Singh et al., 1991). The Mesoamerican pool is considered to be ancestral to the Andean pool, and there is evidence of a bottleneck in genetic diversity occurring years ago during the establishment of the Andean pool (Bitocchi et al., 2012).

Common bacterial blight (CBB) is a major foliar and seed-borne disease affecting bean production throughout the world. The disease is caused by the pathogen *Xanthomonas axonopodis* pv. phaseoli (*Xap*, for review see Hayward and Waterston, 1965; Starr, 1981; Leyns et al., 1984; Buell, 2002; Yun et al., 2006; Perry and Pauls, 2012). The pathogen is endemic in the soil of most regions where dry beans are cultivated, and the ability of the pathogen to colonize the seeds of infected plants greatly increases the risk of pathogen carry-over from year to year.

Immunity to *Xap* has not been observed in *P. vulgaris*, but complete resistance has been observed in members of the secondary and tertiary gene pools such as *Phaseolus coccineus* (Park and Dhanvantari, 1987; Miklas et al., 1994) and *Phaseolus acutifolius* (for review see Park and Dhanvantari, 1987; Singh and Schwartz, 2010). OAC-Rex (Parker, 1985; Scott and Michaels, 1992; Michaels et al., 2006), HR45 and HR67 (Park and Dhanvantari, 1994) were developed from interspecific crosses between *P. vulgaris* and *P. acutifolius*. In particular, OAC-Rex, which was registered as the first bacterial blight resistant cultivar in Canada in 2002 (Michaels et al., 2006), derives its resistance from the *P. acutifolius* line PI 440795. Resistance loci have been associated with molecular markers *Pv*-CTT001 (GI:169360), SU91 (GI:156072919), BNG71 and BNG21 in OAC Rex (Tar'an et al., 2001; Shi et al., 2012; Durham et al., 2013).

The breeding lines HR67 and HR45 were derived from XAN 159, which was developed from a cross between ICI Piajo and *P. acutifolius* PI 319443 (Parker, 1985). Two major CBB resistance Quantitative Trait Loci (QTL) associated with the markers UBC420 and SU91 have been identified in these lines (Yu et al., 2004). Epistatic interaction between the SU91- and UBC420-associated QTL has been observed. When only one marker is present, the resistance is mild to moderate in the field, but when both are present, strong resistance is observed (Shi et al., 2011; Durham et al., 2013).

In addition to the *P. acutifolius*-derived CBB resistance, some *P. vulgaris*-derived resistance was found in lines descended from the Great Northern landrace that is associated with the SAP6 marker (GI:218137924) on chromosome 10 (for review see Gepts et al., 2008). Although the level of resistance provided by this QTL is low, it appears to act epistatically with SU91 (Vandermark et al., 2008), indicating that similar interactions may be occurring between gene products of SU91, SAP6, and UBC420. In contrast, epistatic interactions have not been observed between SU91 and *Pv*-CTT001 (Durham, 2011).

Although the development of molecular markers has aided research on CBB resistance in beans, and facilitated the selection of new CBB resistant varieties, previously the lack of genome sequence information for bean has hindered the identification of the resistance genes associated with the markers. In contrast with many other crop plants, genomic resources for *P. vulgaris* were relatively limited before the release of the genomic sequence of the Andean breeding line G19833 (*P. vulgaris* v1.0, DOE-JGI and USDA-NIFA, http://www.phytozome.net/commonbean). Prior to September 2012, there were just over 148,000 ESTs listed in GenBank along with a limited number of molecular markers (Ramirez et al., 2005). Schuleter et al. (2008) sequenced the ends of 41,717 BAC clones from a BAC library of the G19833 line. At the time, it was the most significant collection of *P. vulgaris* sequence information, including over 62 Mb of sequence data and accounting for ~9.54% of the total *P. vulgaris* genome. In addition, 1536 randomly selected BAC clones were fully sequenced using a shotgun method. The sequence information derived from the end-sequencing and the shotgun sequencing was analyzed using the basic local alignment search tool (BLAST) and compared to sequence data from *A. thaliana*, *M. truncatula*, and *L. japonicus*. These comparisons indicated that approximately 49.2% of the bean genome is composed to repetitive sequence and only 29.3% of the genome contains functional genes. The breakdown of the genic regions was remarkably similar between the shotgun sequenced clones and the BAC end sequences, with approximately 38% of the genes coding for proteins involved in cellular processes and 33% involved in metabolic processes. The sequence information from the shotgun sequencing was more useful for predicting the functions of genes, due to the longer reads, but the low cut-off levels used as part of the assembly and annotation process limited the ability of this study to accurately predict gene function (for review see Gepts et al., 2008; Schuleter et al., 2008).

Comparative genomics in plants is still a relatively new field, and has been limited by the number of completed genome sequences available. Previous studies examining the consequences of interspecific hybridization have largely used molecular markers to quantify the degree of introgression in given populations, predominantly between cultivars and their wild relatives (Arnold et al., 2003; Riesberg et al., 2003; Gill et al., 2011; Andrew et al., 2012; Hufford et al., 2013). SNP marker studies in *Zea mays*, *Pinus pumila*, and *Helianthus petiolaris*, have indicated that hybridization rates can vary along chromosomes. Direct comparisons of gene content in plants have been generally limited to model organisms such as *Arabidopsis thaliana*, as part of the 1000 Arabidopsis genomes project (Cao et al., 2011; Schneeberger et al., 2011). Work involving leguminous species has centered on *Glycine max*, and comparative genetic analyses between the Williams 82 genome, and the LD09-15087a breeding line was used to highlight changes in the *Rhg*1 QTL, which is involved in resistance to *Heterodera glycines*. This study was able to identify resistance genes based on copy number variation and expression studies of resistant and susceptible cultivars (Cook et al., 2012).

With the release of the V1.0 G19833 sequence, genomic comparisons are now possible between the *P. vulgaris* genotypes. This genome was primarily assembled using the Roche 454 platform (Wheeler et al., 2008), combined with Sanger end sequencing of a bacterial artificial chromosome library of G19833. The V1.0 release is covers a total of 521.1 Mb, arranged in 708 scaffolds, and 41,391 contigs, covering all 11 bean chromosomes. *In-silico* gene annotation has resulted in 27,197 loci, containing 31,638 presumptive genes. The genomic sequence is available for download at www.phytozome.net.

Genomic sequencing of OAC-Rex is currently ongoing, with initial assemblies yielding contigs in the megabase range that collectively account for 83% of the G19833 sequence (DiNatale et al., unpublished results). The two datasets represent one of the first opportunities to compare large genomic regions between *P. vulgaris* genotypes and the first to compare the G19833 genome with a line that has an interspecific cross in its pedigree.

The goal of the current study is to compare the genes in the regions surrounding the *Pv*-CTT001, SU91 and SAP6 markers for CBB resistance candidates between OAC-Rex and G19833. The assumption is that although the order, orientation, and identity of most of the gene annotations are likely to be conserved between the two genomes, the OAC-Rex will genome will have some unique genes that will be of interest for their possible involvement in CBB-resistance. In addition, the comparison should provide insights into the effects of introgression genome structure.

## Materials and methods

### DNA isolation and sequencing

Genomic DNA was isolated from intact nuclei using a modified protocol of Zhang et al. (1995), Leaf tissue (10 g) was frozen in liquid nitrogen and ground to a fine powder using a mortar and pestle. The ground leaf tissue was divided into two 5 g aliquots and placed into 50 ml Oakridge tubes with 100 ml of ice-cold 1 × Homogenization Buffer (HB; 0.01 M Trizma base, 0.08 M KCl, 0.01 M EDTA, 1 mM spermidine-HCl, 1 mM spermine-HCl + 0.15% β-mercapthoethanol + 0.5% Triton X-100 + 2% PVP-40; [Sigma, St. Louis MO]). The samples were gently mixed with a glass rod until homogeneous. Following a 20 min incubation on ice, during which the samples were swirled every 2 min, they were filtered through 2 layers of cheese cloth and one layer of Mira cloth to remove large debris.

The tubes were centrifuged at 1800 g for 20 min at 4°C and the supernatant was discarded. The pellets were washed three times with cold Wash Buffer (WB; 1XHB, 0.5 M sucrose, 0.5% Triton X-100) and were resuspended with a fine paintbrush in the tube. For the first wash, an additional 30 ml of WB was added to the homogenate, and the samples filtered through two layers of sterile cheese cloth and one layer of Mira cloth. The tubes were centrifuged at 1200 g for 20 min at 4°C and the supernatant discarded. For further washes (5 were required), only 5 ml of buffer WB were used, and the homogenate was not filtered prior to centrifugation. During the washing steps the color of the pellet changed from green to white. For the final wash, the two tubes were combined together into a total volume of 10 ml. Buffer WB was added to a final volume of 30 ml and the samples were centrifuged at 1200 g for 20 min The pellets were resuspended in 0.5 ml of buffer HB (50 μl/g tissue) using a fine paint brush and placed on ice.

The intact nuclei were processed using a Qiagen DNeasy Plant Kit (Qiagen, Valencia CA). The samples were processed according to the manufacturer's instructions. After the final ethanol wash, the samples were resuspended in 600 μl of TE buffer (10 mM Tris pH 8.0 and 1 mM EDTA).

The DNA was sequenced using the Illumina HiSeq platform at the Center for Applied Genomics, Toronto, Ontario. Two mate pair libraries, 2.5 and 7 kb were prepared using the manufacturer's protocol for 50 bp read lengths. Shotgun sequencing using paired-end, 100 bp reads was performed to give an overall sequence depth greater than 100 (DiNatale et al., unpublished results).

Scaffolding and contig assembly was performed at the University of Windsor using SOAP2 *de novo* and ABYSS. For details regarding the assembly and annotation of the OAC-Rex genome, please refer to our companion publication “Assembly and annotation of a short-read draft genome sequence for *P. vulgaris* cv. OAC-REX” (DiNatale et al., Unpublished results).

### Nucleotide analyses

Sequences from G19833 and OAC-Rex containing the markers of interest were identified through BLAST analysis using BioEdit (http://www.mbio.ncsu.edu/bioedit/bioedit.html). For the SNP markers (Table 1), the only nucleotide variation permitted was at the polymorphism site, and all other nucleotide identities must be conserved (120/121 bp). For the *Pv*-CTT001, SU91 and SAP6 markers, an *e*-value of 0.00 was required for the match to be considered valid.

**Table 1:** **BeanCAP Marker locations in G19833**.

**Marker**	**Designation**	**Location (bp)**	**Strand**
sc01018ln115966_84234_C_T_313074185	M-4-1	33248 … 33368	−
BARC-PV-0000055	M-4-2	42209 … 42902	+
sc01018ln115966_70654_C_T_313060605	M-4-3	45967 … 46087	−
sc00518ln214431_147554_A_G_234673221	M-4-4	261113 … 261233	−
sc00518ln214431_114746_C_T_234640413	M-4-5	297639 … 297758	−
sc00518ln214431_82881_A_G_234608548	M-4-6	328728 … 328848	−
BARC-PV-0004479	M-4-7	340663 … 341029	+
sc00518ln214431_38754_C_T_234564421	M-4-8	370459 … 370579	−
sc00112ln569344_96989_A_G_97815989	M-4-9	1085963 … 1086083	−
sc00112ln569344_110476_T_C_97829476	M-4-10	1099451 … 1099571	−
sc00187ln435150_255274_G_A_135407497	M-8-1	59250063 … 59250183	−
sc00187ln435150_224424_G_A_135376647	M-8-2	59280917 … 59281037	−
sc00187ln435150_172862_C_T_135325085	M-8-3	59331499 … 59331619	−
sc00187ln435150_154478_T_C_135306701	M-8-4	59349712 … 59349832	−
sc00187ln435150_76559_G_A_135228782	M-8-5	59423268 … 59423388	−
sc00187ln435150_54957_C_T_135207180	M-8-6	59444877 … 59444997	−
sc01730ln60519_17555_C_T_372768027	M-8-7	59615745 … 59615865	+
sc00051ln745799_70203_A_G_57666871	M-10-1	39971947 … 39951128	+
sc00051ln745799_128693_T_C_57725361	M-10-2	39980908 … 40008959	+
sc00051ln745799_135759_T_G_57732427	M-10-3	39984666 … 40016026	+
sc00051ln745799_252780_G_A_57849448	M-10-4	40199812 … 40131518	+
sc00051ln745799_324665_A_C_57921333	M-10-5	40236338 … 40202342	+
sc00051ln745799_372713_A_G_57969381	M-10-6	40267427 … 40250278	+
sc00051ln745799_385474_T_G_57982142	M-10-7	40279362 … 40263040	+
sc00051ln745799_432491_C_A_58029159	M-10-8	40309158 … 40309932	+
sc00051ln745799_449711_C_T_58046379	M-10-9	40456276 … 40326797	+
sc00051ln745799_476649_T_C_58073317	M-10-10	41024662 … 40352254	+
sc00051ln745799_537307_C_T_58133975	M-10-11	40191957 … 40410943	+
sc00051ln745799_568762_G_A_58165430	M-10-12	40222811 … 40445901	+
sc00051ln745799_626273_T_G_58222941	M-10-13	40273393 … 40501692	+
sc00051ln745799_666408_G_A_58263076	M-10-14	40291606 … 40541562	+
sc00051ln745799_673318_T_C_58269986	M-10-15	40302924 … 40548477	+
sc00051ln745799_726997_G_T_58323665	M-10-16	40365162 … 40603487	+
sc00309ln320131_21655_C_A_179959800	M-10-17	40386771 … 40734400	+
sc00309ln320131_95334_A_G_180033479	M-10-18	40557639 … 40804315	+
sc00309ln320131_169758_T_C_180107903	M-10-19	39951008 … 41010466	+
sc00309ln320131_184806_A_G_180122951	M-10-20	40008839 … 41026660	+

### Sequence annotation and genomic comparisons

The sequence data was examined for open reading frames using FGENESH (http://www.softberry.org), with the *Medicago* gene model. The predicted gene sequences were BLASTed to the NCBI nucleotide and EST databases. Predicted protein sequences were compared using tBLAST. All comparisons were made using the CLC Genomics Workbench built-in BLAST functionality. Significant homologies were defined by having *E*-values <*E*^−30^ for nucleotide comparisons and *E*-values <*E*^−20^ for amino acid sequences, and were plotted onto the assembled contigs.

Functional characterization of predicted genes was accomplished by examining the resulting BLAST homologies, and categorizing them into one of 8 groups based on previously characterized genes (Schuleter et al., 2008): 1. Unknown/No Homology, 2. Hypothetical Predicted Protein, 3. Defense/Signaling/Stress, 4. Hormone Response, 5. Transcription/Translation Factor, 6. Structural Component, 7. Metabolism and 8. Transposable Element. In the event of two predicted functions, the result with the highest homology was generally selected with the exception of BLAST homologies to BAC clones, or other genomic survey sequence results, as these do not represent functional gene characterizations.

Syntany analyses were conducted in CLC Genomics Workbench using the integrated BLAST functionality with an internal database containing the predicted cDNA sequences. The sequences were compared between the G19833 and OAC-Rex lines only within a single chromosome range. Homologies were considered significant with *E*-values <*E*^−50^, or when nucleotide homology was greater than 90% for short gene calls, defined by CDS sequences less than 300 bp.

Protein analyses were performed using the predicted mRNA sequences from FGeneSH, and translated into amino acid sequences using the CLC Genomics Workbench standard model. Protein domains were predicted using the Pfam database (Finn et al., 2010). Transmembrane domains were predicted using the TMHMM web server (Krogh et al., 2001; http://www.cbs.dtu.dk/services/TMHMM/).

### Genomic resources

Genomic sequence data for the unannotated V1.0 G19833 release was obtained from Phytozome (McClean et al., Unpublished Results; http://www.phytozome.net/).

Sequences for the BeanCAP single nucleotide polymorphism (SNP) markers was obtained with the assistance of Dr. Perry Creegan (USDA, Beltsville Agricultural Research Center, MD, USA).

Genomic sequence data for OAC-Rex will be available from the Applied Bean Genomics and Bioproducts website (http://www.beangenomics.ca).

## Results

### QTL and contig identification

**Figure 1:**
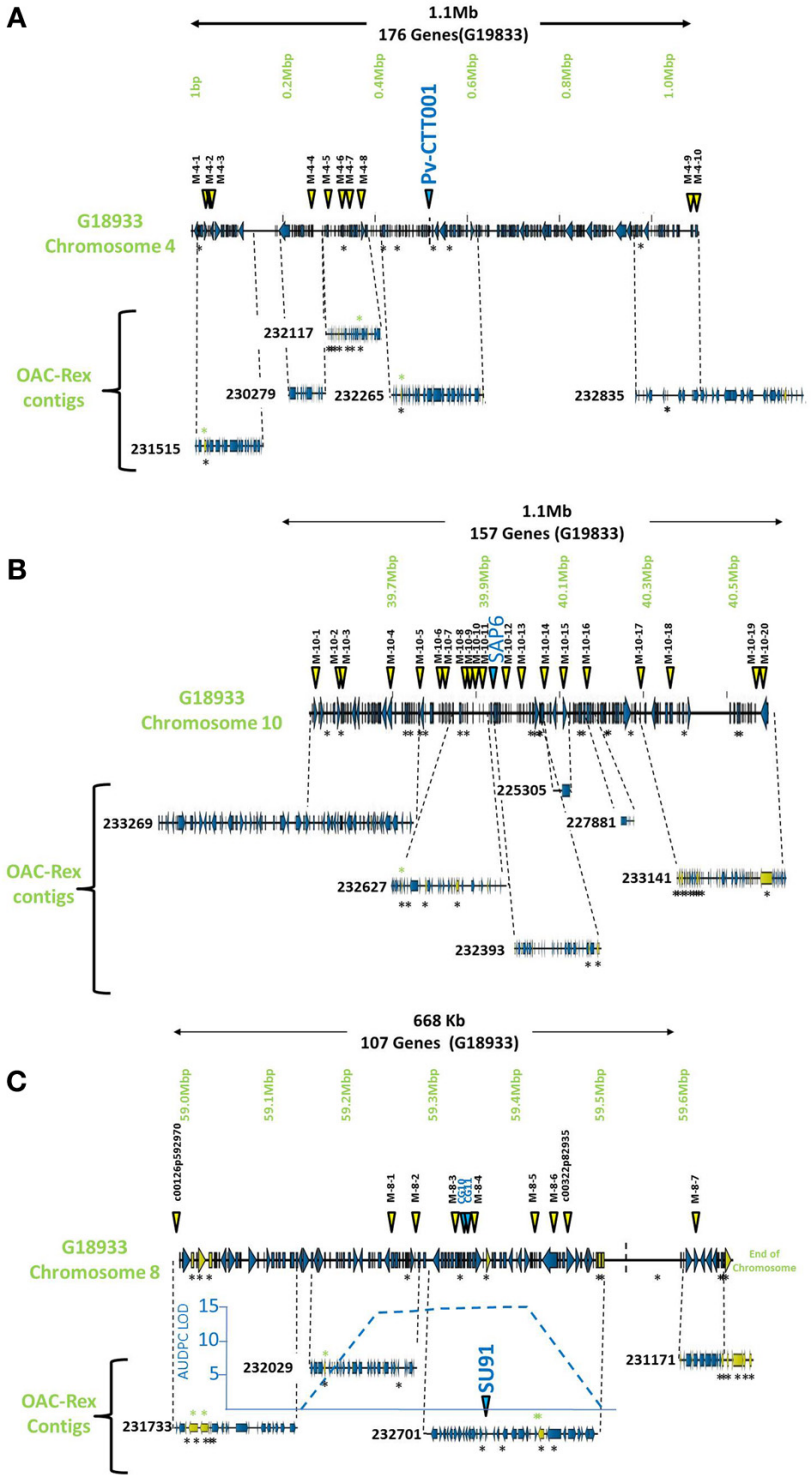
**Comparison of genomic regions in G19833 and OAC-Rex surrounding the CBB-associated molecular markers.** The OAC-Rex contigs are positioned relative to the G19833 sequence from chromosome 4 **(A)**, 10 **(B)**, and 8 **(C)**, encompassing the *Pv*-CTT001, SAP6, and SU91 markers, respectively. The locations of *Pv*-CTT001, SAP6, and SU91 are denoted by purple triangles, while the locations of the SNP markers are indicated with yellow triangles. The locations of unique genes are indicated by the presence of black asterisks (^*^) under the sequences. Unique genes from OAC-Rex with homology to *P. acutifolius* ESTs are indicated by green asterisks above the contigs. For the chromosome 8 figure, the positions of two additional gene-based markers (CG10 and CG11, Shi et al., 2012) are also denoted by purple triangles. Additionally, the location of the identified CBB-QTL from the region surrounding the SU91 marker in a OAC-Rex X Sanilac population is indicated by the embedded chart, normalized to the markers on chromosome 8 (Xie et al., unpublished results).

The pseudochromosome sequences from G19833 were interrogated with the sequences for the molecular markers *Pv*-CTT001, SAP6 and SU91. The *Pv*-CTT001 marker was found at the beginning chromosome 4 in G19833, starting at 517,577 bp and ending at 517,741 bp (Figure 1A). The SAP6 marker was found on chromosome 10 in G19833, starting at 39,938,699 bp and ending at 39,939,569 bp (Figure 1B). In both cases, the marker sequences only had one significant homology in the genome assembly for G19833. The SU91 marker was not found in the G19833 sequence. However, a SU91-associated region in the G19833 sequence was selected using the c00126p592970 and c00322p82935 markers, which were previously mapped to the SU91 QTL in HR45 (Figure 1C, Xie et al., unpublished results; Shi et al., 2012). It starts at 58,994,870 bp and ends at 59,444,870 bp. The SU91 marker was previously identified in a 450 kb region on chromosome 8 in HR45, which is flanked by the markers c00126p592970 and c00322p82935 (Xie et al., unpublished results). A larger region, from the c00126p592970 marker to the end of chromosome 8, corresponding to a 666 kb fragment, was selected from the G19833 sequence (58,994,870–59,662,532 bp) for further comparisons.

**Figure 2:**
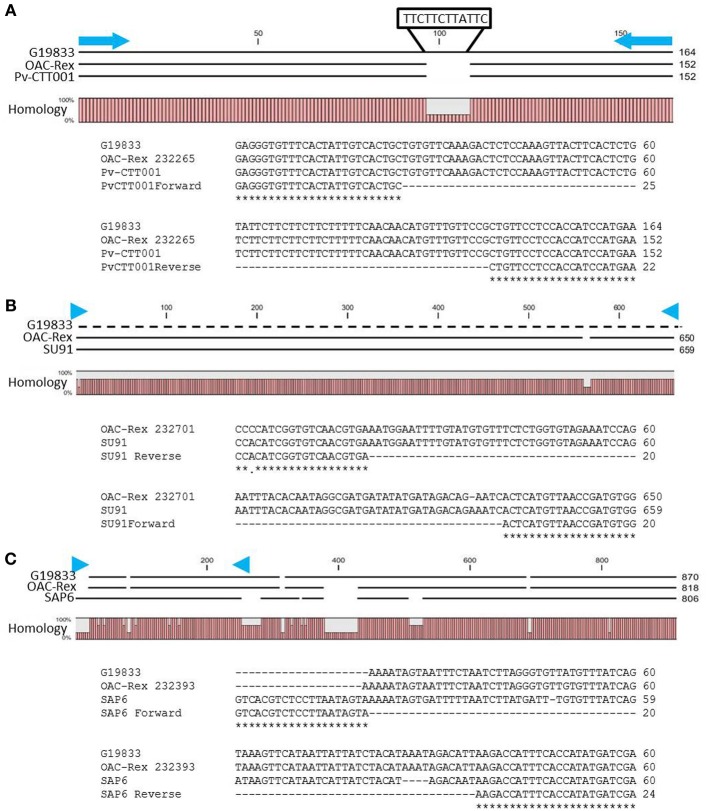
**Alignment of genomic DNA sequences associated with CBB-resistance markers; (A) *Pv*-CTT001, (B) SU91, and (C) SAP6.** The degree of conservation of the genomic sequences in G19833 and OAC-Rex between the marker PCR primers is given. The G19833 fragment amplified by the *Pv*-CTT001 would be expected to be 12 bp larger than the OAC-Rex sequence because of the insert noted **(A)**. For the SU91 marker **(B)**, a null sequence is provided for the G19833 sample, as the marker is not present in this line. The locations of the PCR primers used to amplify these fragments are indicated by blue arrows. ClustalW alignments of the primer regions are given for G19833 and OAC-Rex. For the SAP6 marker **(C)**, both G19833 and OAC-Rex lack the majority of the forward primer sequence, but they contain the majority of the sequence associated with the SCAR marker, including the reverse PCR primer.

The genomic region covered by the *Pv*-CTT001 marker was highly conserved in G19833 and OAC Rex lines, but the sequence that would be amplified from the marker primers in G19833 would be expected to be 12 bp longer than from the OAC Rex sequence because of the presence of four additional CTT repeats, starting at 517633bp, in the former (Figure 2A).

The SU91 marker was not found in the G19833 sequence (Figure 2B), and interestingly, although the G1833 line does not have the sequence corresponding to the forward primer for the SAP6 marker, it does contain the reverse primer sequence and the adjacent sequences are very similar between OAC Rex and G19833 (Figure 2C). The location of the SAP6 fragment in G19833 and OAC-Rex is in the same region as has been described for lines derived from the Great Northern #1 Sel 27 (for review see Miklas et al., 2003; Singh and Schwartz, 2010).

Once the markers in G19833 were identified, larger sections of the chromosomes (667.7 kb to 1.1 Mb) were selected to account for the estimated recombination distances between the markers and potential resistance genes (for review see Perry and Pauls, 2012). These regions were examined for other markers and gene sequences that could be used to identify contigs in the OAC-Rex assembly. In particular, the regions were annotated using FGENESH, and populated with SNP markers from the BeanCAP collection (Hyten et al., 2010; Perry Creegan, Personal Communication) (Figure 1; Table 1).

The G19833 chromosome 4 fragment was found to contain 171 genes and 10 SNP markers, covering the full length of the fragment (Figure 1A) giving an annotated gene density of 0.16 genes/kb. Interestingly, the SNP markers were heavily skewed toward the beginning and end of the selected region, with 8 of the SNPs occurring in the first 370 kb of the fragment, and the last 2 found starting at 1.09 Mb. The intervening region was found to contain only the *Pv*-CTT001 marker at 517,577 bp, with no additional landmarks found in the latter half of the selection.

The chromosome 10 fragment contained 130 predicted genes, with an overall gene density of 0.12 genes/kb (Figure 1B). This was the lowest gene density for the three fragments, although it can partially be explained by the presence of a 100 kb unknown nucleotide region, starting at 910 kb. The 20 SNP markers were located across the whole region, with only limited clustering of two markers near the beginning of the fragment.

The fragment from chromosome 8 contained 105 genes and 7 SNP markers (Figure 1C). The gene density of 0.16 genes/kb is similar to that found for the other fragments. As with the chromosome 4 fragment, the distribution of the SNP markers was somewhat skewed, with the markers being evenly distributed starting 250 kb into the fragment. Only the c00126p592970 marker was found at the start of the region, with no other landmarks present until the M-8-1 SNP marker located at 59,248,128 bp. The terminal end of the fragment was also found to contain a long region of unknown nucleotides (N) spanning approximately 150 kb (59,511,657 bp to 59,661,425 bp), which limited the comparisons that could be made in this region.

The markers identified in the three regions were used to interrogate the K41 assembly of the OAC-Rex sequence (www.beangenomics.ca), and yielded 16 contigs, ranging from 21.1 to 476.8 kb in size. The chromosome 4 region was populated by 5 OAC-Rex contigs (231515, 230279, 23117, 232265, and 232835), covering a total distance of 701 kb, and containing 114 genes. Because contig 232835 extends beyond the range of the 1.1 Mb fragment; only 100 of these genes were contained within the G19833 region examined. Four of the contigs, 231515 (105,837 bp), 230279 (67,213 bp), 232117 (139,956 bp) and 232265 (150,801 bp), were aligned sequentially along the first 615,105 bp of the G19833 sequence and contained the first 9 SNP markers. The *Pv*-CTT001 marker was found in contig 232265. All the contigs, with the exception of 230279, contained multiple markers, which facilitated their positioning along the G19833 sequence. The 237,587 bp OAC-Rex contig 232835 contained the remaining 2 SNP markers and aligned with the terminal end of the G19833 sequence.

The region surrounding the SAP6 marker on chromosome 10 was homologous to 6 OAC-Rex contigs, namely: 233269 (476,805 bp), 232627 (195,818 bp), 232393 (165,172 bp), 225305 (21,147 bp), 227881 (36,196 bp), and 233141 (359,578 bp). They contained 160 genes. However 42 genes were found outside of the G19833 region on contigs 232269 and 233141. All of the contigs contained multiple SNP markers, (2 and 6 markers per contig).

The region surrounding the SU91 marker was populated by 4 contigs, 231733 (117,016 bp), 232029 (133,816 bp), 232701 (207,708 bp) and 231171 (91,949 bp), covering a total distance of 550 kb and containing 96 predicted genes. Contigs 231733 and 231171 contained single SNP markers, c00126p592970 and M-8-7, respectively; while the remaining contigs each contained between 2 to 4 markers. No contigs were found within the region from 59,511,657 to 59,661,425 bp, due to the presence of a large number of unknown (N) nucleotides (*P. vulgaris* v0.9, DOE-JGI and USDA-NIFA, http://www.phytozome.net/commonbean). A previous characterization of a 65 kb HR45 BAC contig library (32H6), containing the SU91 marker resulted in the development of additional molecular markers (CG10 and CG11) for this region (Shi et al., 2012; Figure 1C) that were used to identify the corresponding region in G19833 and OAC-Rex (G19833 c8:59,333,799-59,396,160 and 232701:94178-160157 bp, respectively).

### Gene content

**Figure 3:**
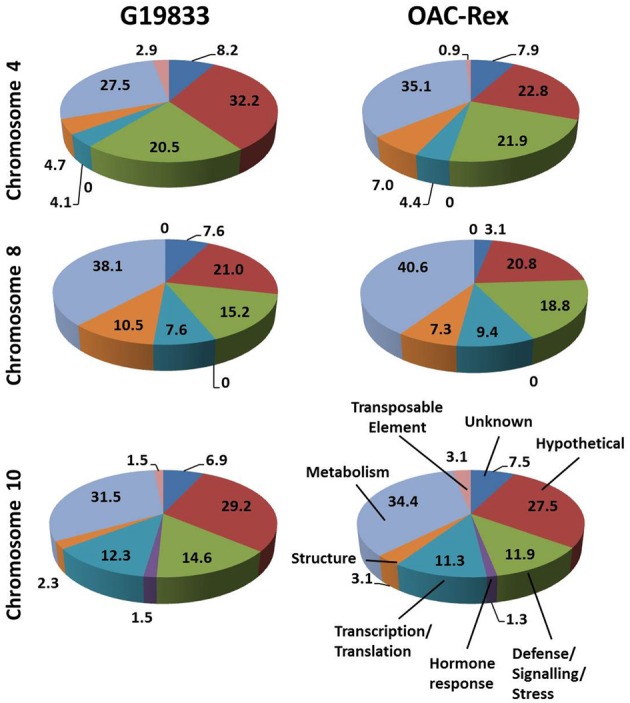
**Distributions of annotated genes in the CBB-marker associated regions among functional categories.** The genes from each region were BLASTed against the NCBI nucleotide, EST and protein databases, then categorized into 8 categories: Unknown/No Match; Hypothetical Protein; Defense/Signaling/Kinase; Hormone Response; Transcription/Translation Factor; Structural; Metabolism and Transposable Element. The percentage for each category are listed on in the figure.

The predicted genes in the three CBB marker-associated regions for the OAC-Rex and G19833 lines were grouped into functional categories for comparison (Figure 3). The annotations from chromosome 4 of the G19833 line indicated that the largest category of the predicted genes fell into the Unknown or Hypothetical protein categories; accounting for 8.2 and 32.2% of the total, respectively. Among the genes to which a specific roles could be ascribed, those functioning in Metabolism and Defense/Signaling/Stress constituted the two largest categories, with 27.5 and 20.47% of the total, respectively. The remaining genes coded for a collection of structural components and transcription/translation regulators (4.7 and 4.1%) and a small number (2.9%) of transposable elements.

A similar gene distribution was found among the 5 contigs from OAC-Rex associated with *Pv*-CTT001. Genes falling into the Unknown and Hypothetical categories accounted for 30.7% of the total. As in the G19833 line, Metabolism and Defense/Signaling/Stress genes were the largest groups of genes with identifiable function, making up 35.1 and 21.9% of the total, respectively. Similar to G18833, the remaining genes were structural genes (7.0%), transcription/translation factor genes (4.4%), and transposable elements (0.9%).

The gene annotations for the chromosome 8 segment associated with SU91 in G19833 indicated that general Metabolism genes accounted for the single largest category, with 38.1% of the total gene content. This was followed by the Unknown or Hypothetical genes, with a combined total of 28.6%. The remaining genes were categorized as Defense/Signaling/Stress, Transcription/Translation and Structural related, and accounted for 15.4, 7.6, and 10.5% of the total, respectively. The groupings were very similar for the genes in the OAC-Rex contigs associated with SU91. Specifically the distribution among categories was: 40.6% Metabolism, 23.9% combined Unknown and Hypothetical, 18.8% Defense/Signaling/Stress related, 4.1% Transcription/Translation factors, and 7.3% Structural genes.

The region from chromosome 10 of line G19833 had Unknown or Hypothetical genes accounting for 36.1% of the total. This value was lower in the OAC-Rex chromosome 10 contigs associated with SAP6 (35%), however, it still represented the largest group of genes in the identified OAC-Rex contigs. The values for other categories of genes were also comparable between the two lines. The number of Metabolism and Defense/Signaling/Stress genes in OAC-Rex was 34.4 and 11.9%, respectively, compared to 31.5 and 14.6%, respectively for G19833. There was little variability seen among the other gene categories between the gene annotations in the G19833 and OAC-Rex fragments.

### Comparison of chromosome gene organization

**Figure 4:**
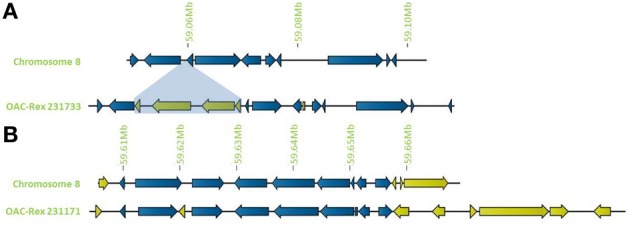
**Distribution of unique genes in G19833 and OAC-Rex. (A)** Comparison of chromosome 8 showing the insertion of a cluster of unique genes around 59.06 Mb and a single unique gene at 59.08 Mb. **(B)** Comparison of a section of chromosome 8 showing positions of unique genes in G19833 and OAC-Rex at the end of chromosome 8. Unique gene clusters in both G19833 and the OAC-Rex contig 231171 were found, with 3 unique genes and 6 unique genes, respectively.

In addition to general gene content, the physical gene order and orientation were well-conserved between G19833 and OAC-Rex over all three chromosome regions. Where differences did occur, it was generally not in the form of large spans containing multiple consecutive genes, but instead unique genes were found interspersed between conserved gene regions, normally with no more than 3 unique genes in tandem, and covering regions of less than 15,000 bp. Exceptions to this were found on chromosome 8 (Figure 4), with two clusters found. The first was at the start of contig 231733 encompassing 4 CC-NBS-LRR genes from 10135 to 33238 bp. The second was found on the terminal end of chromosome 8, where a cluster of 6 additional genes on OAC-Rex contig 231171 were found when compared to G19833.

Outside of these regions, the novel genes found in G19833 and OAC-Rex were interspersed over most of the annotated regions within generally conserved genetic content. Of the 171 genes annotated for the G19833 sequence from chromosome 4, 68 were not found in OAC-Rex contigs. Due to the absence of any markers or unique gene features between 621,481 and 974,996 bp, no corresponding OAC-Rex contigs were found within this region. A reference assembly of this region using the raw shotgun reads from OAC-Rex indicated that OAC Rex has homologous sequences covering 69% of the 60 genes that are present in the G19833 line in this region, but they do not coalesce into a single contig and are consequently not shown. Also, it is not possible to positively conclude that the OAC Rex sequences detected in this analysis are from this region and not another region of the OAC-Rex genome. Outside of this region, only 7 unique genes were present in G19833. Unique genes were also present in OAC-Rex, with 11 annotated genes having no analogue in the G19833 line (Table 2). Many of these genes could be categorized as Defense/Signaling/Kinases. However, only 3 genes (231515-4-018, 232117-4-26, and 232265-4-003) were found to have homology to *P. acutifolius* EST accessions from a drought stressed *P. acutifolius* library (LIBEST_026709, VSU-ARS-L15; Narina and Bhardwaj, unpublished).

**Table 2:** **Unique genes identified in OAC-Rex**.

**Name**	**NCBI accession**	**Location (bp)**	**Gene size (bp)**	**CDS (bp)**	**Most significant BLAST homology**	**Most significant BLAST homology**	***P. acutifolius* homology**
231515-4-018	KF429149	88211 … 91461	3251	849	356527439	Uncharacterized protein (*G. max*)	HO78729
232117-4-003	KF429150	12855 … 13403	549	345	357487113	Hypothetical protein	None
232117-4-006	KF429151	21348 … 22425	1078	387	357442399	Self-incompatibility protein (*M. truncatula*)	None
232117-4-008	KF429152	31950 … 32549	600	477	357442403	Hypothetical protein	None
232117-4-009	KF429153	33379 … 34447	1069	438	357442399	Self-incompatibility protein (*M. truncatula*)	None
232117-4-010	KF429154	39424 … 40023	600	477	357518591	Self-incompatibility protein (*M. truncatula*)	None
232117-4-013	KF429155	47342 … 47916	575	306	357442399	Self-incompatibility protein (*M. truncatula*)	None
232117-4-026	KF429156	107677 … 109514	1838	312	139387442	Ribosomal protein S4 (*P. vulgaris*)	HO794491
232265-4-003	KF429157	14978 … 17004	2027	1866	356573349	Cytochrome P450 86B1-like (*G. max*)	HO796932
232835-4-025	KF429158	208950 … 209520	571	456	357518591	Self-incompatibility protein (*M. truncatula*)	None
232835-4-026	KF429159	210079 … 213362	3284	1983	353685479	Putative retrotransposon (*P. vulgaris*)	None
231733-8-003	KF429192	10135 … 11344	1210	810	357493209	CC-NBS-LRR resistance protein (*M. truncatula*)	None
231733-8-004	KF429160	13860 … 22394	8535	4929	357493209	CC-NBS-LRR resistance protein (*M. truncatula*)	HO797873
231733-8-005	KF429161	24691 … 31932	7242	4269	357460465	NBS/LRR resistance protein-like protein *(M. truncatula)*	HO792495
231733-8-006	KF429162	32042 … 33238	1197	822	357493209	CC-NBS-LRR resistance protein (*M. truncatula*)	None
231733-8-010	KF429163	46759 … 47309	551	327	356552149	*Medicago* pentatricopeptide repeat	None
232029-8-004	KF429193	17858 … 20060	2203	1320	351723325	*G. max* chalcone reductase	HO786606
232029-8-017	KF429164	91187 … 91947	761	378	359806707	Uncharacterized protein LOC100816276 (*G. max*)	None
232701-8-007	KF429165	42491 … 59132	16,642	2970	356553425	Niemann PicC1	HO791620
232701-8-008	KF429166	62281 … 69187	6907	1293	356553425	Niemann PicC1	HO801643
232701-8-016	KF429167	113984 … 114536	553	129	396582350	EGF-like 1 protein (*P. vulgaris*)	None
232701-8-020	KF429168	128102 … 130242	2141	342	396582345	Putative binding protein	None
232701-8-021	KF429169	132737 … 133012	276	113	396582360	RING/U-box domain-containing protein (*P. vulgaris*)	None
231171-8-001	KF429170	2535 … 5496	2962	615	218437660	Hypothetical protein	None
231171-8-002	KF429171	9776 … 12987	3212	786	260753266	Hypothetical protein	None
231171-8-003	KF429194	13119 … 25074	11,956	1689	267753266	Hypothetical protein	None
231171-8-004	KF429172	25436 … 26688	1253	336	270342091	Hypothetical protein	None
231171-8-005	KF429173	30962 … 33200	2239	432	260753266	Hypothetical protein	None
231171-8-006	KF429174	37134 … 39836	2703	525	None	None	None
232627-10-008	KF429175	17887 … 19809	1923	705	87162935	Ribonuclease H	HO799029
232627-10-016	KF429176	102766 … 104102	1337	372	None	Unknown	None
232627-10-018	KF429195	109610 … 114230	4621	972	356558954	Cation/calcium exchanger 4-like	None
232627-10-021	KF429177	149640 … 149815	176	126	312059257	Hypothetical protein	None
232393-10-016	KF429178	118287 … 119145	859	330	356522402	Hypothetical protein	None
232393-10-020	KF429179	156523 … 159933	3411	312	None	Unknown	None
233141-10-002	KF429180	6415 … 11763	5349	3366	51854301	Putative polyprotein	None
233141-10-003	KF429181	13594 … 20654	7061	4101	353685479	Putative retrotransposon	None
233141-10-004	KF429182	24619 … 25453	835	378	356522390	Suppressor of npr-1	None
233141-10-006	KF429196	31169 … 33542	2374	636	356558721	Putative disease resistance protein	None
233141-10-007	KF429183	37228 … 39059	1832	1023	356558721	Putative disease resistance protein	None
233141-10-011	KF429184	64258 … 65884	1627	708	356522390	Suppressor of npr-1	None
233141-10-012	KF429185	66591 … 71982	5,392	708	120529942	Hypothetical protein	None
233141-10-013	KF429186	73306 … 74730	1,425	570	356522390	Suppressor or npr-1	None
233141-10-014	KF429187	74786 … 76925	2,140	354	356522390	Suppressor or npr-2	None
233141-10-033	KF429188	240188 … 242639	2,452	285	255640830	Hypothetical protein	None
233141-10-034	KF429189	246548 … 248053	1,506	555	312034114	Hypothetical protein	None
233141-10-036	KF429190	256995 … 257918	924	384	357509013	Hypothetical protein	None

On the SAP6-associated fragments from chromosome 10, 30 genes from G19833 were not found in the OAC-Rex contigs. The 5 contigs from OAC-Rex spanned the entire region, with only 11 genes located in regions not covered by OAC-Rex contigs. Interestingly, 18 genes from the OAC-Rex contigs were unique (Table 2). Although the Defense/Signaling/Kinase category only accounted for 11.9% of the overall gene content in OAC-Rex, they made up 33% of the unique gene content. Only a single unique gene was found to have homology to the *P. acutifolius*, with 232627-10-008 having homology to HO799029. Gene order and orientation was less conserved between OAC-Rex and G19833 for the SAP6 associated regions, when compared to the regions from chromosome 4 and 8.

The sequence surrounding the SU91 marker on chromosome 8 encodes 105 genes in line G19833, with a region between 59,511,657 and 59,661,425 bp that was mostly Ns. This prevented the identification of corresponding contigs from OAC-Rex to populate this region, although most of the G18933 region is covered with 4 OAC-Rex contigs. Overall, there were 12 unique genes in G19833 and 18 unique genes in OAC-Rex (Figure 1C, Table 2). In OAC-Rex, no function could be ascribed to 7 of these genes, however, 11 genes did have homology to previously characterized or predicted proteins, including: 4 resistance gene candidates (231733-8-003, 231733-8-004, 231733-8-005, and 231733-8-006), a pentatricopeptide repeat (231733-8-010), a chalcone reductase (232029-8-004), two Niemann Pick like gene (232701-8-007 and 232701-8-008), an EGF1-like gene (232701-8-016), and a RING-U-box domain containing protein (232701-8-020).

**Figure 5:**
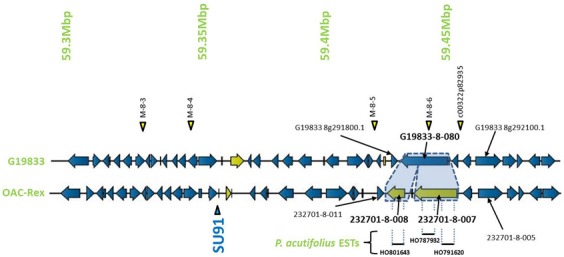
**Comparison of the regions surrounding the SU91 marker in OAC-Rex contig 232701 with the corresponding sequence from G19833 chromosome 8.** Unique genes are marked in yellow, and the two genes (232701-8-007 and 232701-8-008) that are homologous with the G19833 Niemann Pick transporter gene are highlighted for comparison. 232701-8-007 has homology with two *P. acutifolius* ESTs (HO787932 and HO791620) while 232701-8-008, has homology to a single *P. acutifolius* (EST, HO801643). Highly conserved genes bordering this region in G19833 and OAC-Rex are labeled. The location of molecular markers are indicated with triangles above and below the sequence.

**Figure 6:**
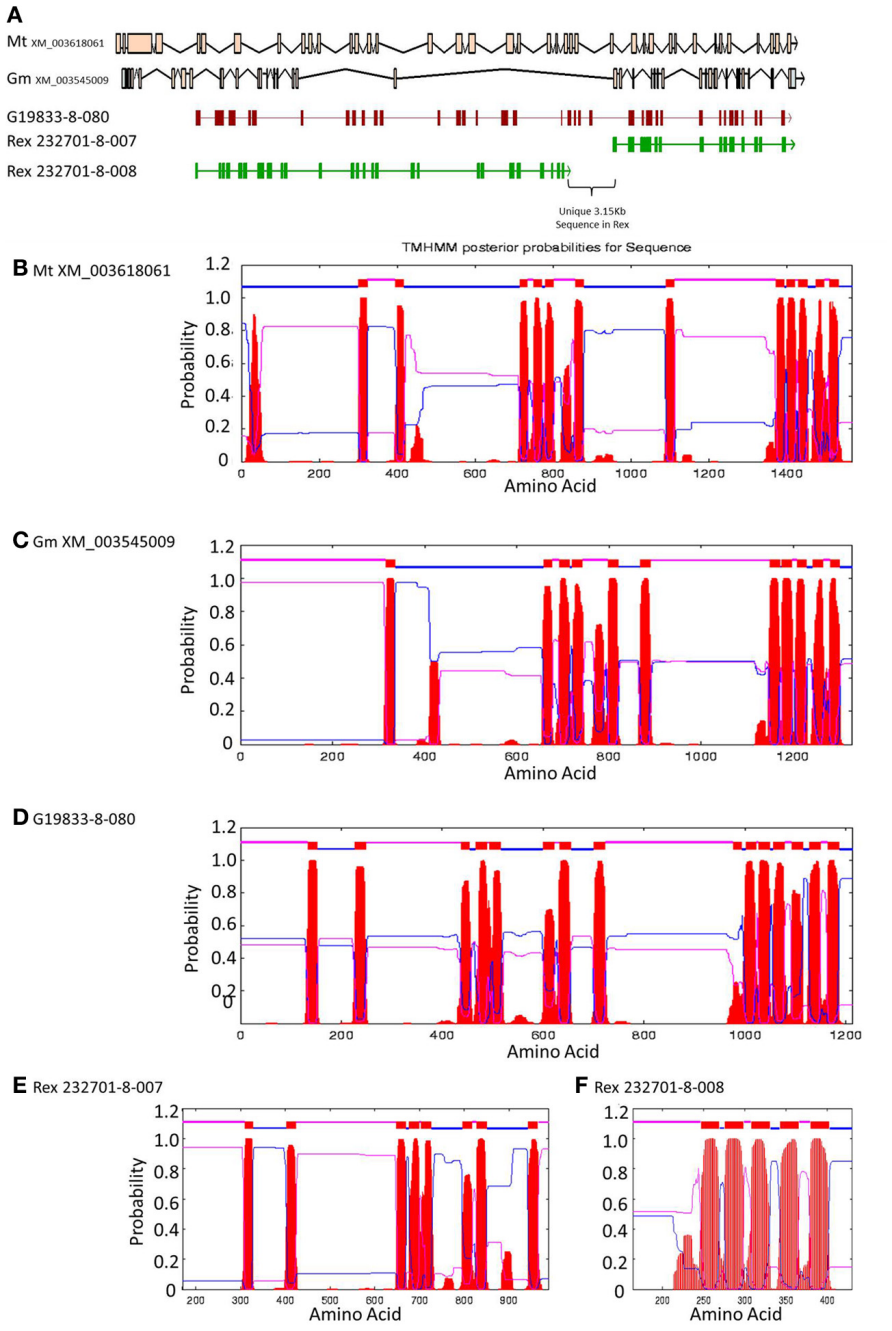
**Comparisons of Niemann Pick-like genes from *G. max*, *M. truncatula*, and G19833 *P. vulgaris* and genes in the same locus in OAC-Rex. (A)** The exon/intron structures of the genes. Secondary structure probabilities determined by TMHMM Server v. 2.0 for *M. truncatula* **(B)**, *G. max* **(C)**, G19833 **(D)**, OAC-Rex gene 232701-8-007 **(E)**, and OAC-Rex gene 232701-8-008 **(F)**.

Encompassed by the QTL for CBB-resistance the OAC-Rex sequence appeared to contain a mutation/rearrangement of gene G19833-8-80 in G19833, which is homologous to a Niemann Pick cholesterol transporter from *Homo sapiens* (Carstea et al., 1997). It appears that this gene is split into two genes in OAC-Rex. Both genes in OAC-Rex (232701-8-007 and 232701-8-008) had homology to the Niemann Pick cholesterol transporter. An alignment of these genes relative to the G19833-8-080 and Niemann Pick-type genes from *G. max* and *M. truncatula* (Figures 5, 6A), showed that the OAC-Rex genes appear to have been derived from different regions of the G19833-8-080 gene, with 232701-8-007 representing the region from the N-terminus to 900 aa, and 232701-8-008 representing the 1100 aa to the C-terminus. The intervening space between these two genes is over 3000 bp in length in the OAC Rex genome. Both genes have stop codons and polyA signals.

**Figure 7:**
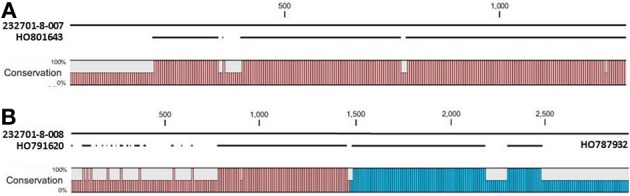
**Alignments of *P. acutifolius* ESTs with the predicted CDS sequence from the Niemann Pick locus in OAC-Rex.** The percent conservation between three accessions from a drought stressed *P. acutifolius* library (Narina and Bhardwaj, unpublished) and the OAC-Rex genes 232701-8-007 **(A)** and 232701-8-008 **(B)** are shown. EST HO801643 contained a stop codon at the same position as the OAC-Rex gene, neither HO0791620 nor HO787932 were complete and did not contain start or stop codons. Their positions in the alignment are denoted by red and blue bars in the Conservation graph, respectively.

The OAC-Rex Niemann Pick-like genes were also homologous to *P. acutifolius* ESTs. Gene 232701-8-008 was highly homologous to HO801643 (Figure 7A), an EST from LIBEST 026709, a *P. acutifolius* library derived from drought-stressed leaf tissue from line VSU-ARS-L15. The match covers 81% of the predicted gene sequence, with 1052 out of 1239 bp conserved. The homology for 232701-8-007 was not as clear, as no singe EST covered the entire length of the gene. Rather, two ESTs, HO791620 and HO787932 (Figure 7B), were found to be homologous to over half of the predicted gene sequence, with an overall homology of 99% in these regions. The ESTs do not appear to be complete, as they both lack start and stop codons (for review see Nakamoto, 2009).

**Figure 8:**
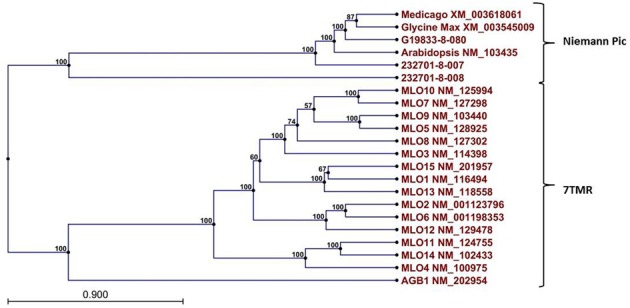
**Dendrogram of relationships among Niemann Pick and 7 transmembrane receptor (7TMR) proteins from *P. vulgaris*, *M. truncatula*, and *A. thaliana*.** The dendrogram was constructed with CLCGenomics Workbench v. 5.0. Numbers indicate the bootstrap value for each node.

A Pfam domain search of both of the Niemann Pick-like genes from OAC-Rex, as well as the gene from G19833 showed that the two OAC-Rex genes appear to encompass separate regions of the G19833, *G. max* and *M. truncatula* genes (Figure 6; XM_003545009 and XM_003618061, respectively). The 232701-8-007 gene encodes a protein with 8 transmembrane domains and contains a sterol sensing domain, while 232701-8-008, encodes a protein containing 5 transmembrane domains, but no other identifiable domains. The transmembrane domains shared some homology to the 7-transmembrane (7TM) domain proteins from Arabidopsis and *Hordeum vulgare*, which have been associated with susceptibility to fungal and bacterial pathogens through the *MLO* genes in previous studies (Piffanelli et al., 2004; Consonni et al., 2006). Analysis of the predicted proteins with the 7TMRminer program (Lu et al., 2009; http://bioinfolab.unl.edu/emlab/7tmr), indicated that none of the *P*. *vulgaris* proteins have exact matches to this motif. In particular, neither of the OAC-Rex genes contained the required 7 transmembrane domains, while the gene from G19833 was found to contain too many (12 potential transmembrane domains identified). But the genes did have some characteristics in common with 7TMR proteins, indicated by positive matches for the non-alignment-based comparisons [Support Vector Machine (SVM) –amino acid, SVM-dipeptide and partial least squares regression-auto/cross-covariance], However, these comparisons are not considered to be as strong support for the 7TMR motif as the alignment-based comparisons (Lu et al., 2009). The similarity of the predicted proteins from *P. vulgaris* to 7TMR proteins from Arabidopsis (AGB1) and barley (MLO1-15), and Niemann Pick proteins from Arabidopsis, *G. max*, and *M. truncatula* was also examined using nearest neighbor analysis. The resulting dendrogram showed that the genes from *Phaseolus* cluster more strongly with the Niemann Pick proteins than 7TMR proteins (Figure 8). However, the relationship is weakest for the OAC-Rex genes.

**Figure 9:**
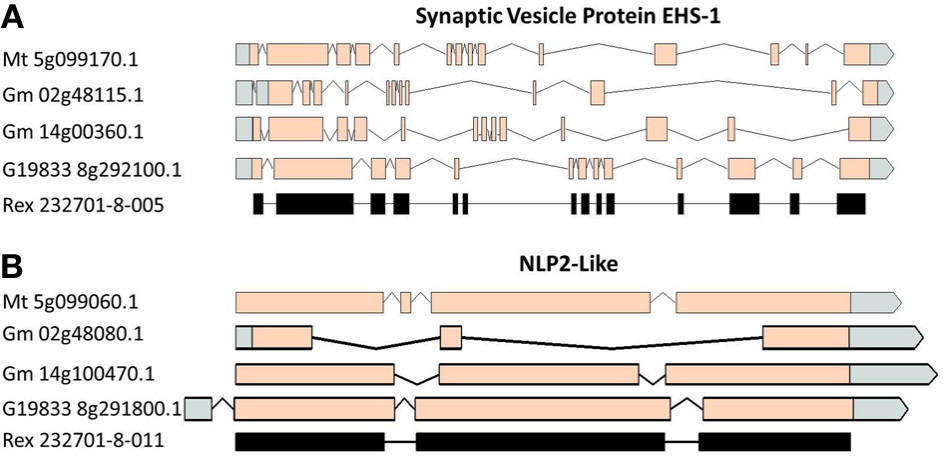
**Gene structure comparisons of genes surrounding the Niemann Pick gene locus in *M. truncatula*, *G. max*, and *P. vulgaris*.** Two genes from upstream **(A)** and downstream **(B)** of the Niemann Pick like genes are compared for their exon/intron structure similarities. Homologies were very high, BLAST *e* = 0.0.

A comparison of the exon/intron structures of the *P. vulgaris* Niemann Pick-like genes to those in other species indicated that, although the exon structure between the OAC-Rex genes and the G19833 gene are highly conserved, the structures are only marginally conserved between G19833 *P. vulgaris*, *G. max*, and *Medicago*. A BLAST analysis indicated that there is only one copy of the Niemann Pick gene in G19833, three in *G. max* and one in *Medicago* (data not shown). An analysis of the surrounding genes in G19833 and OAC-Rex (Figure 9) indicates that the exon structure is conserved across species in these genes, and that only the Niemann Pick genes seem to possess this divergent exon/intron structure. Although expression data from P. vulgaris was not available at this time, an examination of the Medicago Gene Atlas (Benedito et al., 2008; http://mtgea.noble.org/v3/) indicated that the Niemann Pick analogues from *M. truncatula* (Medtr5g099070.1) and *G. max* (Glyma14g00400.1 and Glyma02g48070.1) appear to be expressed in all indexed tissues (data not shown).

The role of EGF1-Like genes (232701-8-016) in plants is poorly understood, although there is some evidence that they are related to wall associated protein kinases (WAKs; Wagner and Kohorn, 2001; Verica and He, 2002). However, the small size of the annotated gene in *P. vulgaris* (Gene: 553bp, CDS: 129 bp), would indicate that this gene does not encode a functional protein (Silva and Goring, 2002). No corresponding gene analogue was found in G19833.

**Figure 10:**
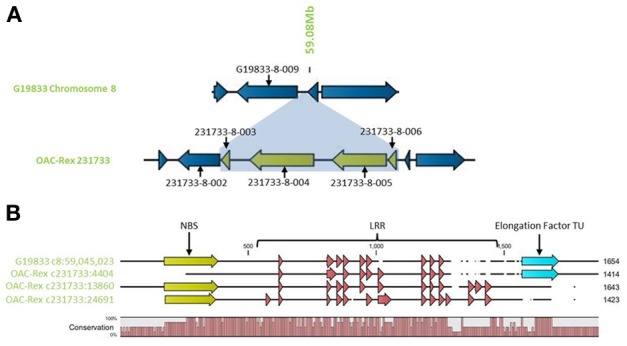
**Comparison of the resistance gene candidates from G19833 chromosome 8 and OAC-Rex contig 231733. (A)** An alignment of the G19833 region containing G19833-8-009 with the OAC-Rex region containing a homolog 231733-8-002 showing an insertion containing a unique gene fragment and two complete R-gene analogs 231733-8-004 and 231733-8-005. **(B)** Alignments of the predicted protein products of OAC-Rex genes 231733-8-002, 231733-8-004, and 231733-8-005 with the G19833-8-009 gene. The level of conservation among the 4 genes is indicated by the Conservation bar graph. The positions of nucleotide binding sequences (NBS), leucine rich region (LRR) domains, and the Thermal Unstable (TU) elongation factor domains are shown on the sequence.

Outside of the mapped resistance QTL, 5 genes in OAC-Rex from the 231733 contig (231733-8-002, 231733-8-003, 231733-8-004, 231733-8-005, and 231733-8-006) were found to have homology to coiled coil-nucleotide- binding site-leucine rich repeat (CC-NBS-LRR) resistance genes from *M. truncatula*, although the shortness of genes 231733-8-003 and 231733-8-006 (at 1210 and 1197 bp, respectively) suggest that they may not be functional resistance genes. The region containing the four genes, 231733-8-003, 231733-8-004, 231733-8-005, and 231733-8-006, appears to represent an insertion event in OAC-Rex, after 231733-8-002 and corresponding to the region after G19833-8-009 in G19833 (Figure 10A). Genes 231733-8-002, 231733-8-004, and 231733-8-005 were homologous to *P. acutifolius* ESTs (HO792495, HO797873, and HO792495, respectively).

Only gene G19833-8-009 in G19833 was found to be homologous to the CC-NBS-LRR type resistance genes in this region, and an alignment of putative proteins encoded by G19833 G19833-8-009 and genes 231733-8-002, 231733-8-004, and 231733-8-005 from contig 231733 (Figure 10B) showed that all 4 genes are closely related. A Pfam domain search indicated that G19833 G19833-8-009 and OAC-Rex 231733-8-002 were the most closely related. Both had 9 LRR domains arranged in a very similar pattern. OAC-Rex 231733-8-004 and 231733-8-005 have 12 and 13 LRR domains, respectively, with a significantly different distribution compared to 231733-8-002 and G19833 G19833-8-009. All four genes appear to share a common motif of 5 LRR regions starting at approximately 700 aa, although the positions and spacing's of these repeats vary from gene to gene. The greatest level of variability was found in the C-terminal region, where OAC-Rex 231733-8-004 and 231733-8-005 possess several additional LRR regions when compared to G19833 G19833-8-009.

In addition to the LRR domains, a nucleotide binding site was found in the N-terminal regions of G19833 G19833-8-009, OAC-Rex 231733-8-004, and 231733-8-005. This feature was not found in OAC-Rex 231733-8-002, although the overall homology between all the genes in this region is over 90%.

## Discussion

### Gene content and orientation are conserved between G19833 and OAC-Rex

Although the current study compared genomic regions in beans of Mesoamerican and Andean origin, and the former included an interspecific cross with *P. acutifolius* in its pedigree, the overriding finding was that there is a high degree of similarity in overall gene identity, order, and orientation between OAC-Rex and G19833. This similarity is particularly striking because the regions that were chosen for the comparison were thought to be different because they are associated with resistance to CBB in OAC-Rex, introduced through introgression, and there is no report of resistance in G19833. In fact, all three regions associated with the markers for CBB resistance were largely conserved between the two lines, even in the case of the SU91 marker, whose sequence is almost entirely absent from the G19833 sequence. These results indicate that, even in regions where molecular markers associated with specific introgression events occur, the overall frequency of unique gene transfer is low. This was true for the regions that had unique (SU91, *Pv*-CTT001) and shared (SAP6) markers. Although the susceptibility of genomic regions to introgression varies along chromosomes (Andrew et al., 2012; Hufford et al., 2013), the amount of new genetic material, including unique genes will most likely still be low, even in regions with high levels of hybridization as assayed by dense marker screens. To identify unique genes, it appears that direct sequence comparisons across large genomic regions are needed to accurately assess the introgression of new genes into genotypes. In plants derived from more recent hybridization events, percentage of new material may be higher (Baack and Rieseberg, 2007), but in a cultivated variety, such as OAC-Rex, which was backcrossed several times to *P. vulgaris* lines, the unique gene content is relatively low.

We also found that the unique gene content generally consisted of individual or paired genes, interspersed among the conserved regions. However, exceptions to this did occur in chromosomes 8, which had larger gene clusters containing four or more unique genes. In the case of the cluster at the end of G19833 chromosome 8, and in contig 231171 in OAC-Rex, the unique genes represent the end of the annotated chromosome in both regions, and no homologies could be found for any of these genes. The other unique gene cluster in OAC-Rex coincided resistance gene loci in contig 231733. Resistance gene clusters are known to undergo genomic duplication and rearrangement at higher frequencies than for most other regions of the genome (Meyers et al., 1998; Kim et al., 2009). Thus, it is possible that the unique genes in OAC-Rex in this regions may be the result of independent genetic rearrangements, rather than be signatures of introgression. However, the observation that the unique resistance genes were highly homologous to *P. acutifolius* ESTs in this region supports the suggestion that the unique genes were obtained by introgression from the interspecific parent (*P. vulgaris* × *P. acutifolius*) in the OAC-Rex pedigree. However, the presence of genes with homology to *P. acutifolius* ESTs is not conclusive evidence for an insertion event, especially for resistance genes, as some of the resistance gene analogs in G19833 were also homologous to *P. acutifolius* genes.

Generally, no function could be ascribed to the many of the unique genes identified in this study, with 31% of the annotated genes having no known function. However, they remain interesting targets for future analyses because of the possibility that they are of interspecific origin that may bring new traits to the cultivated dry bean. Additional data, perhaps from future transcriptional profiling, may provide insight into the biological roles of these genes.

### Identifying potential CBB-resistance genes in OAC-Rex

Resistance derived from interspecific crosses in plants could arise through a number of mechanisms of gene modification associated with introgression, including: elevated transposable element activity (Liu and Wendel, 2000), altered gene methylation (Xiong et al., 2013), insertional mutagenesis (Hegarty and Hiscock, 2004) and novel gene introduction (Gill et al., 2011). Based on the hypothesis that CBB-resistance in OAC-Rex is due to the introgression of *P. acutifolius* DNA into the *P. vulgaris* genome, it is likely that genes unique to OAC-Rex will be responsible for this trait. Examples from the current study would include the two unique genes within the mapped SU91 resistance QTL on chromosome 8, in OAC Rex contig 232701.

The locus that is altered in OAC Rex and contains two genes (232701-8-007 and 232701-8-008) homologous to different portions of the Niemann Pick transporter in the G19833 genome. These two genes in the Niemann Pick transporter locus in OAC Rex could represent a loss-of-function mutation, where the gene function has been lost due to the 3000 bp insertion, or it may represent recombination with genes in *P. acutifolius* that have homology to this gene, but have independent functions. Structurally, the OAC-Rex genes appear to be derived from distinct regions of the G19833-8-080 gene, with the sterol sensing domain contained within 232701-8-007. Both OAC-Rex genes do appear to include a transmembrane domain; however, the traditional sterol sensing and transport role associated with the Niemann Pick transporters may not be preserved (Munkacsi et al., 2007). It is possible that OAC-Rex genes 232701-8-007 and 232701-8-008 have direct roles in defense against *Xap*, and that the deletion of G19833-8-080 in this variety was the result of a recombination event arising from the interspecific cross in its pedigree. Furthermore, the observation that the genes surrounding the Niemann-Pick loci in G19833 and OAC-Rex are homologous and share syntany with genes in other leguminous species (McConnell et al., 2010; Muchero et al., 2011), but the exon structure of the Niemann Pick-like genes in G19833-8-080, and OAC-Rex 232701-8-007, and 232701-8-008 are divergent, relative to the sequences from *G. max* and *M. truncatula*, may indicate that these genes are unique to *P. vulgaris* (Figure 6).

The function of the Niemann Pick transporter (NCP1) in mammalian cells is to transport of cholesterol from biliary micelles after absorption from the intestinal lumen, to the endoplasmic reticulum for conversion to cholesterol esters by acyl-CoA acyltransferase (Garver et al., 2002; Abi-Mosleh et al., 2009). Homologs of NPC1 have been identified across the animal and plant kingdoms (Munkacsi et al., 2007). In Arabidopsis, two Niemann Pick gene homologues have been identified, AT1G42470.1 and AT4G38350.1. Both of the proteins encoded by these genes have been localized to various regions of the cell, including: the plasma membrane (Benschop et al., 2007; Mitra et al., 2007), the vacuolar membrane (Carter et al., 2004; Jaquinod et al., 2007a,b) and the plasmodesmata (Fernandez-Calvino et al., 2011) Although there are difference in gene structure (Figure 6), given the degree of sequence conservation observed among the Niemann Pick genes across plant species it is likely that similar subcellular distributions occur for the Phaseolus Niemann Pick gene. But it is important to note that Niemann Pick gene function has not been studied in any plant species to date, and most of the functional analyses have focused on mammalian species but the conservation of the sterol binding motif in the G19833 Niemann Pick gene (and the 232701-8-007 OAC-Rex gene) suggest that they are also involved in sterol transport (Jang et al., 2000; Carter et al., 2004; Mitra et al., 2007). The complexity of phytosterol synthesis, compared to animal cholesterol synthesis, may account for the broad distribution of the Niemann Pick protein in plant membranes.

Given that there appears to only be one copy of the Niemann Pick gene in *P. vulgaris*, its disruption in OAC-Rex would be expected to knock out the function from the plant. Given the ubiquitous expression seen in the *M. truncatula* and *G. max* database (Benedito et al., 2008), it is possible that this protein fills a required niche in the cell. However, OAC-Rex does not exhibit any obvious negative phenotype, it can be concluded that the role of the Niemann Pick gene is either not essential for plant development, is accommodated by the action of a related protein, or is being fulfilled by the independent function(s) of the unique genes 232701-8-007 and 232701-8-008. Evidence for the independent function hypothesis is that both genes contain discrete transcription start sites and polyA signals, they are separated by 3 kb of intervening DNA and discrete *P. acutifolius* EST sequences homologous to both of the OAC-Rex genes have been previously identified in this study. Interestingly, disruption of sterol synthesis in Arabidopsis has been associated with an increase in basal resistance to bacterial infection. In particular, the stimulation of β-sisterol to stigmasterol conversion that is observed in Arabidopsis after infection by *Pseudomonas syringae* has been shown to increase the susceptibility of the plant to infection and inhibition of stigmasterol synthesis (in P450 CYP710A1 mutants) enhances plant resistance to avirulent and virulent *P. syringae* strains (Griebel and Zeier, 2010). The authors suggest that the plasma membranes, altered by the increased stigmasterol content, may have different pathogen perception or defense signaling properties. Perhaps if Niemann Pick gene(s) in G19833 and OAC-Rex play roles in sterol sensing, sterol transport/synthesis, and/or cell signaling pathways though brassinosteroids they are coupled to basal resistance in plants (for review see Clouse, 2002). Whatever their particular molecular role(s) are in plants, the example of the increase in resistance associated with the inhibition of stigmasterol synthesis, through the disruption of gene function, represents an interesting model for how an introgression event might result in increased resistance to CBB in beans.

The potential for these genes to represent non-traditional R-gene candidates is supported by previous studies involving *G. max* and *H. vulgare*. Comparative genomic analysis of the *Rhg*1 QTL in the *H. glycines*-resistant breeding line LD-09-15087a to the published Williams 82 genome indicated that copy number variation in genes from the *rhg*1-b interval were responsible for the resistance trait in the breeding line. The actual role of the genes is unknown at this time, but the authors hypothesized that the proteins are involved in intercellular transport in the roots (Cook et al., 2012).

In *H. vulgare*, another interesting model for non-traditional R-gene mediated resistance has been studied involving the involving 7TMR proteins. Previous research on a 7TMR gene from barley, called MLO (Jørgensen, 1992), showed that the disruption of this gene is associated with resistance to powdery mildew in barley and Arabidopsis, and also to resistance to bacterial pathogens, notably *Pseudomonas syringae*, *Xanthomonas campesteris* pv. *vesicatoria* and other type III secretions system-possessing bacteria in *Capsicum annuum* and Arabidopsis (Kim and Hwang, 2012; Lewis et al., 2012). In these systems the MLO encoded proteins are thought to repress the transport of material(s) required for papilla construction at the site of pathogen contact with the plant cell wall. When the functional allele is present, the transport of PENETRATION 2 (PEN2) and VESSICLE-ASSOCIATED MEMBRANE PROTEIN 722 (VAMP722) through PENETRATION 3 (PEN3) and the PENETRATION1/SYNAPTOSOMAL-ASSOCIATED PROTEIN 33 (PEN1/SNAP33) complex (Piffanelli et al., 2004) are prevented. Plants with a mutation in *mlo* are unimpeded in building a physical barrier against infection, and as a result, have a greater level of resistance to the pathogen. Given the taxonomic similarity between *Xanthomonas* and *Pseudomonas* genera (De Ley et al., 1966), it is reasonable to propose that similar resistance mechanisms could protect plants against both pathogens. Although the genes in G19833 or OAC-Rex do not appear to be close homologues for *MLO*, a similar mechanism may be operative in which replacement or modification of a gene required for pathogenicity is altered by introgression, thus allowing a basal defense response in OAC Rex.

The EGF-like proteins, like the one found in OAC-Rex (232701-8-016), are thought to play a role in cell wall expansion in plants, and are part of the wall associated kinase family (WAKs). In Arabidopsis, 22 WAKs have been identified, and they have the unique ability to convey signals from the cell wall to the cytosol (He et al., 1999; Silva and Goring, 2002). Additionally, WAK1 has been directly associated with pathogen defense against *P. syringae* in Arabidopsis (He et al., 1998). In *wak*1 null mutants, infection with *P. syringae maculicola ES4326* was lethal, and systemic acquired resistance did not function. Conversely, plants with a functional WAK1 survived infection. WAKs have a Ser/Thr kinase, an extracytoplasmic domain (ectodomain) with several EGF-like repeats that are thought to bind to Oligogalacturonides (OGs) released from the plant cell wall as a result of pathogen attack (Hendrickson et al., 2000). However, the gene in OAC Rex is much smaller than the Arabidopsis isoforms, with a predicted CDS of only 129 bp, as compared to AT3G52850.1 with a CDS of 1872 bp. Comparisons with other *WAK* genes from *Oryza sativa* did not show any significant homology to previously reported genes, thus indicating that this gene annotation does not encode for a functional protein (Silva and Goring, 2002). A similar comparison of the G19833 sequence from the same region did not indicate the presence of any *EGF* or *WAK* genes (data not shown).

In addition to the unique genes found within the mapped QTL, the 2 unique R-Gene candidates in Contig 231733, 232733-8-004, and 232-733-8-005, may also contribute to CBB-resistance in OAC Rex (Ellis et al., 2000). Although these resistance gene candidates are outside the main QTL for the resistance trait on chromosome 8, it is known that CBB-resistance is conditioned by several interacting loci (Gepts et al., 2008). At best, the major QTL usually account for 20–40% of the variation for resistance (Shi et al., 2012) leaving the possibility that more loci with small additive effects contribute to the full resistance observed in OAC Rex. The propensity of resistance gene clusters to contain unique genes in OAC-Rex, as seen in chromosome 8 (Figures 4, 10) could represent one mechanism for the introgression of new genetic material from an interspecific hybridization. The conservation of resistance gene features, combined with their tendency to occur in multi-gene clusters increases the likelihood for homologous recombination (Michaelmore and Meyers, 1998). When combined with a strong selective pressure from the breeding program, CBB-resistance in the case of OAC-Rex, the resistance gene clusters maintain a higher proportion of unique genes after interspecific hybridization. It is not known if these unique genes will play a direct role in disease resistance in OAC-Rex, but their conservation after backcrossing would indicate at worst a neutral contribution, and more likely a selective advantage (Barton, 1979; Baumgarten et al., 2003; Baack and Rieseberg, 2007; Gill et al., 2011).

The ability to compare genetic content, when combined with accurate mapping data can facilitate the discovery of the genetic sources of agronomic traits. In this study, two non-R-Gene candidates for CBB resistance were identified from Contig 231171 in OAC-Rex, and two R-gene candidates were discovered from Contig 231733. In the study of Shi et al. (2012), the CBB-resistance QTL was mapped to a 430 kb region and 16 genes from the 32H6 BAC clone were examined to aid in the development of additional markers for the resistance QTL. The locations of the previously identified genes were confirmed in the present study (between 59,344,595 and 59,350,725 bp, data not shown) but the comparisons in this study showed that the genes in this region were conserved between G19833 and OAC-Rex, which reduces the likelihood that they are responsible for the resistance. The current approach, which is based on the identification of unique genes in the resistant variety greatly, reduces the number of gene candidates that would need to be individually assessed for the resistance trait. Future comparisons between G19833, OAC-Rex and *P. acutifolius* accessions are planned and will serve to further identify the regions of genetic introgression. Unfortunately, due to the difficulty in creating transgenics *in P. vulgaris* (Kwapata et al., 2012), it is unknown if current transgenic techniques can be successfully used in OAC-Rex, and further experiments will need to be conducted to determine this. However, examination of these genes in other resistant and susceptible lines will further aid in identifying one of the major genetic sources of CBB resistance in *P. vulgaris*.

## Conclusions

This study represents one of the first large scale comparisons of gene content between *P. vulgaris* lines. Overall gene order and orientation were highly conserved between G19833 and OAC-Rex across the regions examined. Our comparisons were somewhat hampered by the presence of N-containing regions on chromosome 8, and by a lack of genetic markers on chromosome 4, but comparisons for all three regions showed that 85% of the gene content was conserved between the two lines in the same physical space. This value did vary between 80% for the SAP6 fragment on chromosome 10, to 90% for the SU91 fragment from chromosome 8, but conserved regions were generally found in large stretches. Interestingly, unique genes were found interspersed throughout the conserved sequences, with novel genes generally occurring individually or in pairs. Exceptions to this were found in regions associated with high genetic variability, such as the resistance gene clusters on chromosome 8 and 10, but the lack of any large unique regions would indicate that landmarks for interspecific introgression events cannot be inferred by the proportion of novel content alone. The identification of 2 Niemann Pick genes from OAC-Rex contig 232701, and two resistance gene candidates from contig 231733 have shown the potential for comparative genomics to identify novel gene content from hybridizations, and can accelerate the search for unique genes. Future analyses on these gene candidates will be conducted to determine if the genes identified in this study contribute to CBB resistance in OAC-Rex.

The results of this study serve as an important starting point for the examination of gene syntany in *P. vulgaris*. Aside from the direct application of syntenic analyses for identifying genes associated with important agronomic traits, future studies examining genome variation at whole genome levels in addition to localized comparisons of genetic variability within individual genes will provide valuable insights into the evolution of the *P. vulgaris* market classes.

### Conflict of interest statement

The authors declare that the research was conducted in the absence of any commercial or financial relationships that could be construed as a potential conflict of interest.
