# Supplementary material for: A comparison of the molecular organization of genomic regions associated with resistance to common bacterial blight in two Phaseolus vulgaris genotypes
Source: Front Plant Sci. 2013 Aug 29;4:318. doi: 10.3389/fpls.2013.00318 (PMC3756299; doi:10.3389/fpls.2013.00318)
Supplement: Supplementary file 1 [file DataSheet1.DOCX]

Supplementary Table 1: Predicted Gene Calls for G19833 and OAC-Rex

| Chromosome | Name | Location (bp) | Strand | Most significant BLAST (gi) |
| --- | --- | --- | --- | --- |
| 4 | G19833-4-001 | 2063..4739 | + | 356531011 |
|  | G19833-4-002 | 7213..15425 | - | 356495039 |
|  | G19833-4-003 | 15935..16410 | + | None |
|  | G19833-4-004 | 16419..19158 | + | 350495039 |
|  | G19833-4-005 | 20700..26950 | - | 356495039 |
|  | G19833-4-006 | 28834..36155 | + | 356531806 |
|  | G19833-4-007 | 37223..38141 | + | None |
|  | G19833-4-008 | 39159..40694 | - | 356511726 |
|  | G19833-4-009 | 41743..49899 | - | 356511728 |
|  | G19833-4-010 | 52171..65132 | + | 356573375 |
|  | G19833-4-011 | 65226..68704 | - | 363806852 |
|  | G19833-4-012 | 68968..73036 | - | 351721164 |
|  | G19833-4-013 | 75630..78538 | + | 351723747 |
|  | G19833-4-014 | 78751..82266 | + | 356570271 |
|  | G19833-4-015 | 84169..86016 | + | 255580061 |
|  | G19833-4-016 | 86846..87589 | - | 358248072 |
|  | G19833-4-017 | 88884..92271 | - | 363807034 |
|  | G19833-4-018 | 92364..94875 | + | 356511718 |
|  | G19833-4-019 | 95303..99981 | + | 356570131 |
|  | G19833-4-020 | 101327..102440 | + | 356513983 |
|  | G19833-4-021 | 103225..114639 | - | 312039343 |
|  | G19833-4-022 | 183004..184830 | + | 312049917 |
|  | G19833-4-023 | 186654..188053 | + | 312049917 |
|  | G19833-4-024 | 190217..215367 | - | 356513981 |
|  | G19833-4-025 | 219056..221785 | - | 308782391 |
|  | G19833-4-026 | 224416..227290 | + | 356573950 |
|  | G19833-4-027 | 228418..228934 | - | 190553308 |
|  | G19833-4-028 | 229053..229624 | - | None |
|  | G19833-4-029 | 233186..234532 | - | 356562595 |
|  | G19833-4-030 | 234625..237124 | - | 356511712 |
|  | G19833-4-031 | 237170..238185 | + | 356513973 |
|  | G19833-4-032 | 238572..245215 | + | 356513973 |
|  | G19833-4-033 | 245856..246602 | - | 255536847 |
|  | G19833-4-034 | 252011..252481 | + | 356573851 |
|  | G19833-4-035 | 255064..255584 | + | 356573851 |
|  | G19833-4-036 | 256102..258493 | - | None |
|  | G19833-4-037 | 260371..262707 | + | 356573837 |
|  | G19833-4-038 | 263086..265893 | + | 312049971 |
|  | G19833-4-039 | 285800..286364 | - | 357518591 |
|  | G19833-4-040 | 290169..290739 | - | 357442399 |
|  | G19833-4-041 | 292069..292644 | - | 357751591 |
|  | G19833-4-042 | 294009..299441 | - | 357513905 |
|  | G19833-4-043 | 303796..305012 | + | 357518591 |
|  | G19833-4-044 | 311733..312363 | - | 357442399 |
|  | G19833-4-045 | 313090..314481 | - | 357442403 |
|  | G19833-4-046 | 319816..320446 | - | 357440477 |
|  | G19833-4-047 | 321535..322072 | - | 357442403 |
|  | G19833-4-048 | 324480..325500 | - | None |
|  | G19833-4-049 | 325782..326369 | - | 357442399 |
|  | G19833-4-050 | 327161..331880 | - | 356511706 |
|  | G19833-4-051 | 332356..333739 | - | 187433923 |
|  | G19833-4-052 | 335186..335830 | - | 350440477 |
|  | G19833-4-053 | 338793..339390 | - | 357440477 |
|  | G19833-4-054 | 339973..341030 | + | 356511708 |
|  | G19833-4-055 | 343339..346866 | - | 255560946 |
|  | G19833-4-056 | 347071..351357 | + | 356511702 |
|  | G19833-4-057 | 352410..357353 | + | 356513971 |
|  | G19833-4-058 | 357376..362321 | + | 356573355 |
|  | G19833-4-059 | 364127..366576 | - | 356573718 |
|  | G19833-4-060 | 369730..377406 | + | 356573694 |
|  | G19833-4-061 | 377500..379717 | + | 356573694 |
|  | G19833-4-062 | 379738..384159 | - | 356573680 |
|  | G19833-4-063 | 395076..396975 | + | 356513965 |
|  | G19833-4-064 | 410151..411462 | - | 312058086 |
|  | G19833-4-065 | 414587..415518 | + | 356555969 |
|  | G19833-4-066 | 415651..422221 | - | 353685485 |
|  | G19833-4-067 | 424687..428750 | + | 14348622 |
|  | G19833-4-068 | 431280..433415 | + | 312039343 |
|  | G19833-4-069 | 442555..444237 | + | 356519967 |
|  | G19833-4-070 | 445882..447995 | + | 356571700 |
|  | G19833-4-071 | 451043..454527 | - | 312043296 |
|  | G19833-4-072 | 454945..456624 | + | 356511700 |
|  | G19833-4-073 | 458676..460468 | - | 38194929 |
|  | G19833-4-074 | 460640..462319 | + | 356511700 |
|  | G19833-4-075 | 465190..467992 | + | 312043296 |
|  | G19833-4-076 | 468186..469865 | + | 356511700 |
|  | G19833-4-077 | 472685..475044 | - | 312049382 |
|  | G19833-4-078 | 480717..483227 | - | 270342074 |
|  | G19833-4-079 | 489358..490981 | - | 356573349 |
|  | G19833-4-080 | 499277..500751 | - | None |
|  | G19833-4-081 | 500911..504896 | + | 356573349 |
|  | G19833-4-082 | 506110..508312 | + | 356513963 |
|  | G19833-4-083 | 509375..513014 | - | 363807898 |
|  | G19833-4-084 | 513655..515556 | - | 125568 |
|  | G19833-4-085 | 515619..516910 | - | 125568 |
|  | G19833-4-086 | 522788..526723 | - | 356513961 |
|  | G19833-4-087 | 528665..536912 | - | 356513961 |
|  | G19833-4-088 | 538729..552514 | - | 356513961 |
|  | G19833-4-089 | 552554..557026 | - | 356513961 |
|  | G19833-4-090 | 562629..567389 | + | 356511698 |
|  | G19833-4-091 | 568710..575272 | + | 356511699 |
|  | G19833-4-092 | 577869..582334 | + | 270342075 |
|  | G19833-4-093 | 582524..585834 | - | 270342074 |
|  | G19833-4-094 | 586334..588254 | + | 270342073 |
|  | G19833-4-095 | 591327..593172 | - | 312056338 |
|  | G19833-4-096 | 596664..601080 | + | 270342072 |
|  | G19833-4-097 | 607213..614079 | + | 356511690 |
|  | G19833-4-098 | 618135..621480 | - | 312055789 |
|  | G19833-4-099 | 623168..627194 | - | 270342067 |
|  | G19833-4-100 | 628023..628825 | - | 270342066 |
|  | G19833-4-101 | 639179..639791 | - | 308764821 |
|  | G19833-4-102 | 642448..646308 | + | None |
|  | G19833-4-103 | 664410..669248 | - | 312049917 |
|  | G19833-4-104 | 669312..669900 | + | 312049917 |
|  | G19833-4-105 | 673107..677151 | - | 14348622 |
|  | G19833-4-106 | 677199..681190 | - | 212276527 |
|  | G19833-4-107 | 681775..685514 | + | 208333581 |
|  | G19833-4-108 | 689907..693645 | - | 212276551 |
|  | G19833-4-109 | 694790..698829 | + | 254971800 |
|  | G19833-4-110 | 698836..703534 | + | 254971801 |
|  | G19833-4-111 | 706879..712200 | - | 212276592 |
|  | G19833-4-112 | 713615..723145 | - | 353685479 |
|  | G19833-4-113 | 725539..726019 | + | 312055789 |
|  | G19833-4-114 | 726752..732208 | - | 312049917 |
|  | G19833-4-115 | 732687..733984 | - | 312049917 |
|  | G19833-4-116 | 736679..740719 | - | 14348622 |
|  | G19833-4-117 | 741355..743327 | - | 356571309 |
|  | G19833-4-118 | 744602..747128 | + | 353685481 |
|  | G19833-4-119 | 748256..749960 | - | None |
|  | G19833-4-120 | 750534..754379 | - | 270342128 |
|  | G19833-4-121 | 756606..758961 | - | 171670642 |
|  | G19833-4-122 | 759819..760367 | + | 308780455 |
|  | G19833-4-123 | 760725..769608 | - | 270342132 |
|  | G19833-4-124 | 770129..775742 | + | 38194929 |
|  | G19833-4-125 | 775775..782468 | - | 357459173 |
|  | G19833-4-126 | 787287..788572 | + | 312058086 |
|  | G19833-4-127 | 788904..791262 | + | 312058806 |
|  | G19833-4-128 | 791987..795832 | - | 276342069 |
|  | G19833-4-129 | 795907..797155 | + | 312039633 |
|  | G19833-4-130 | 803262..805601 | - | None |
|  | G19833-4-131 | 808565..812688 | - | 353685479 |
|  | G19833-4-132 | 812820..829350 | - | 14348613 |
|  | G19833-4-133 | 832304..834130 | + | 312058086 |
|  | G19833-4-134 | 836383..838588 | - | 192324121 |
|  | G19833-4-135 | 838803..842833 | - | 212276551 |
|  | G19833-4-136 | 842877..843858 | - | 270342132 |
|  | G19833-4-137 | 843961..846438 | + | None |
|  | G19833-4-138 | 846664..849213 | - | 270342132 |
|  | G19833-4-139 | 849836..851588 | - | 212276547 |
|  | G19833-4-140 | 851624..854467 | - | 212276549 |
|  | G19833-4-141 | 856899..870821 | - | 356573333 |
|  | G19833-4-142 | 873704..879357 | - | 14348622 |
|  | G19833-4-143 | 880053..882554 | - | 270342132 |
|  | G19833-4-144 | 883898..889969 | + | 312058086 |
|  | G19833-4-145 | 892499..895493 | - | 356555696 |
|  | G19833-4-146 | 896350..899799 | - | 212276527 |
|  | G19833-4-147 | 899848..901286 | + | None |
|  | G19833-4-148 | 906588..908247 | - | 270342132 |
|  | G19833-4-149 | 908841..909925 | - | 270342132 |
|  | G19833-4-150 | 913981..915991 | + | None |
|  | G19833-4-151 | 919774..945259 | - | 212276551 |
|  | G19833-4-152 | 945432..954923 | - | 22573333 |
|  | G19833-4-153 | 954977..955962 | - | 308796630 |
|  | G19833-4-154 | 961142..962964 | - | 312039343 |
|  | G19833-4-155 | 963546..965829 | + | 312058086 |
|  | G19833-4-156 | 965838..971069 | - | 270342068 |
|  | G19833-4-157 | 972860..973817 | - | None |
|  | G19833-4-158 | 976037..979767 | - | 356513957 |
|  | G19833-4-159 | 982517..993487 | - | 356593333 |
|  | G19833-4-160 | 999039..1000027 | - | 356573331 |
|  | G19833-4-161 | 1006333..1006870 | - | 357518591 |
|  | G19833-4-162 | 1010233..1010770 | - | 357518591 |
|  | G19833-4-163 | 1015098..1019567 | + | 356511672 |
|  | G19833-4-164 | 1019864..1024794 | - | 356513955 |
|  | G19833-4-165 | 1025989..1029548 | + | 356513953 |
|  | G19833-4-166 | 1031711..1033591 | + | None |
|  | G19833-4-167 | 1034530..1037302 | + | 255560905 |
|  | G19833-4-168 | 1053730..1057971 | + | 356511682 |
|  | G19833-4-169 | 1058121..1060888 | + | 363807048 |
|  | G19833-4-170 | 1084502..1090069 | - | 356573321 |
|  | G19833-4-171 | 1094081..1099878 | - | 356573319 |
|  | 231515-4-001 | 761..1850 | - | 356513983 |
|  | 231515-4-002 | 3224..7864 | - | 356570131 |
|  | 231515-4-003 | 8333..10800 | - | 356511718 |
|  | 231515-4-004 | 10958..14380 | + | 255574306 |
|  | 231515-4-005 | 15643..16418 | + | 358248072 |
|  | 231515-4-006 | 17275..19075 | + | 255580061 |
|  | 231515-4-007 | 20630..23705 | - | 356570271 |
|  | 231515-4-008 | 24420..27592 | - | 351723747 |
|  | 231515-4-009 | 30205..34437 | + | 351721164 |
|  | 231515-4-010 | 34484..37992 | + | 363806852 |
|  | 231515-4-011 | 38086..50898 | - | 356573375 |
|  | 231515-4-012 | 54709..60806 | + | 356511725 |
|  | 231515-4-013 | 61216..63379 | + | 356511726 |
|  | 231515-4-014 | 65877..73271 | - | 356531800 |
|  | 231515-4-015 | 76683..82962 | + | 356495039 |
|  | 231515-4-016 | 84471..87245 | + | 356495639 |
|  | 231515-4-017 | 87254..87701 | - | None |
|  | 231515-4-018 | 88211..91461 | + | 356527439 |
|  | 231515-4-019 | 95394..100351 | + | 356495639 |
|  | 231515-4-020 | 102834..105056 | + | 356531011 |
|  | 230279-4-001 | 6101..9014 | - | 356573837 |
|  | 230279-4-002 | 10921..12668 | + | None |
|  | 230279-4-003 | 12741..14099 | - | 356573851 |
|  | 230279-4-004 | 16740..17172 | - | 356573851 |
|  | 230279-4-005 | 23897..24643 | + | 355536847 |
|  | 230279-4-006 | 25284..33112 | - | 356513173 |
|  | 230279-4-007 | 33162..35349 | + | 356511712 |
|  | 230279-4-008 | 35710..37250 | + | 356562595 |
|  | 230279-4-009 | 40069..41411 | - | None |
|  | 230279-4-010 | 41450..41996 | + | 190553308 |
|  | 230279-4-011 | 43125..45952 | - | 356573950 |
|  | 230279-4-012 | 50767..52581 | + | 308782391 |
|  | 230279-4-013 | 54677..65890 | + | 356513981 |
|  | 230279-4-014 | 65899..67008 | - | 312039343 |
|  | 232117-4-001 | 6547..7111 | - | 357518591 |
|  | 232117-4-002 | 10888..11458 | - | 357519591 |
|  | 232117-4-003 | 12855..13403 | - | 357487113 |
|  | 232117-4-004 | 14759..17201 | - | 357513905 |
|  | 232117-4-005 | 19596..20171 | - | 357518591 |
|  | 232117-4-006 | 21348..22425 | + | 357442399 |
|  | 232117-4-007 | 25541..26760 | + | 357518591 |
|  | 232117-4-008 | 31950..32549 | + | 357442403 |
|  | 232117-4-009 | 33379..34447 | - | 357442399 |
|  | 232117-4-010 | 39424..40023 | + | 357518591 |
|  | 232117-4-011 | 40853..41921 | - | 357442399 |
|  | 232117-4-012 | 43010..43547 | - | 357442403 |
|  | 232117-4-013 | 47342..47916 | - | 357442399 |
|  | 232117-4-014 | 48703..53682 | - | 255560950 |
|  | 232117-4-015 | 56643..57288 | - | 357440477 |
|  | 232117-4-016 | 60346..60887 | - | 357440477 |
|  | 232117-4-017 | 61202..62529 | + | 356511708 |
|  | 232117-4-018 | 64901..67951 | - | 363807602 |
|  | 232117-4-019 | 69760..73417 | + | 356511702 |
|  | 232117-4-020 | 74470..79399 | + | 356513971 |
|  | 232117-4-021 | 79444..84401 | + | 356573355 |
|  | 232117-4-022 | 86209..88748 | - | 356573718 |
|  | 232117-4-023 | 91835..99613 | + | 356573694 |
|  | 232117-4-024 | 99707..101923 | + | 356573694 |
|  | 232117-4-025 | 101944..106383 | - | 356573680 |
|  | 232117-4-026 | 107677..109514 | + | 139387442 |
|  | 232117-4-027 | 117468..119715 | + | 356513965 |
|  | 232117-4-028 | 124315..129685 | + | 356555969 |
|  | 232117-4-029 | 129818..139731 | - | 356515310 |
|  | 232265-4-001 | 3056..4415 | - | 312649917 |
|  | 232265-4-002 | 6241..8843 | - | 270342074 |
|  | 232265-4-003 | 14978..17004 | - | 356573349 |
|  | 232265-4-004 | 24064..25538 | - | None |
|  | 232265-4-005 | 27913..31167 | + | 356573349 |
|  | 232265-4-006 | 34915..36813 | + | 356513963 |
|  | 232265-4-007 | 37957..41757 | - | 363807898 |
|  | 232265-4-008 | 42174..45600 | - | 25568 |
|  | 232265-4-009 | 51561..55461 | - | 356558851 |
|  | 232265-4-010 | 57430..65685 | - | 356513961 |
|  | 232265-4-011 | 67504..81296 | - | 353513961 |
|  | 232265-4-012 | 81336..85811 | - | None |
|  | 232265-4-013 | 90051..90740 | + | 356511698 |
|  | 232265-4-014 | 91402..96345 | + | 356511696 |
|  | 232265-4-015 | 106736..111212 | + | 270342075 |
|  | 232265-4-016 | 97409..104151 | + | 270342074 |
|  | 232265-4-017 | 111402..114735 | - | 270342073 |
|  | 232265-4-018 | 115227..117942 | + | 312056338 |
|  | 232265-4-019 | 120224..122115 | - | 270342072 |
|  | 232265-4-020 | 125955..130043 | + | 356511690 |
|  | 232265-4-021 | 135616..143011 | + | None |
|  | 232835-4-001 | 34..2410 | + | 230342065 |
|  | 232835-4-002 | 11397..12001 | + | None |
|  | 232835-4-003 | 15706..18328 | + | 356571299 |
|  | 232835-4-004 | 18372..23862 | - | 357495845 |
|  | 232835-4-005 | 38591..40911 | - | 110931746 |
|  | 232835-4-006 | 42261..42751 | - | 356571295 |
|  | 232835-4-007 | 60135..65001 | - | 77999251 |
|  | 232835-4-008 | 65354..70034 | - | 351724723 |
|  | 232835-4-009 | 80364..81386 | - | None |
|  | 232835-4-010 | 82115..89521 | + | 356511676 |
|  | 232835-4-011 | 92167..98047 | + | 356511678 |
|  | 232835-4-012 | 99419..101739 | + | 356542758 |
|  | 232835-4-013 | 108477..111665 | + | 356564998 |
|  | 232835-4-014 | 113690..118271 | - | 357495783 |
|  | 232835-4-015 | 125070..134593 | - | 356513974 |
|  | 232835-4-016 | 134631..144009 | + | 356573319 |
|  | 232835-4-017 | 148698..154364 | + | 356573321 |
|  | 232835-4-018 | 156684..159418 | - | 363807048 |
|  | 232835-4-019 | 159601..166409 | - | 356511682 |
|  | 232835-4-020 | 180091..184385 | - | 356513951 |
|  | 232835-4-021 | 189088..192912 | - | 356513953 |
|  | 232835-4-022 | 192991..199119 | + | 356513955 |
|  | 232835-4-023 | 199417..204270 | - | 356511672 |
|  | 232835-4-024 | 204672..206806 | - | None |
|  | 232835-4-025 | 208950..209520 | + | 357518591 |
|  | 232835-4-026 | 210079..213362 | - | 353685479 |
|  | 232835-4-027 | 215808..216378 | + | 357518591 |
|  | 232835-4-028 | 220174..222096 | + | 351422399 |
|  | 232835-4-029 | 227386..228404 | + | 356573331 |
|  | 232835-4-030 | 234661..236466 | + | 356573333 |
| 8 | G19833-8-001 | 58995210..58996805 | - | None |
|  | G19833-8-002 | 58999514..59008836 | + | 357493208 |
|  | G19833-8-003 | 59009315..59013048 | - | 356552216 |
|  | G19833-8-004 | 59018034..59027221 | + | 83423282 |
|  | G19833-8-005 | 59030576..59035042 | + | 357495317 |
|  | G19833-8-006 | 59037739..59039741 | + | 16805332 |
|  | G19833-8-007 | 59039767..59041102 | + | 351722267 |
|  | G19833-8-008 | 59042553..59044151 | + | 357495321 |
|  | G19833-8-009 | 59045022..59051787 | - | 357493209 |
|  | G19833-8-010 | 59052799..59054046 | - | None |
|  | G19833-8-011 | 59000818..59062739 | + | 356501763 |
|  | G19833-8-012 | 59062756..59066384 | - | 356552149 |
|  | G19833-8-013 | 59067166..59069147 | + | 229368619 |
|  | G19833-8-014 | 59069163..59070085 | - | 357495337 |
|  | G19833-8-015 | 59078558..59088676 | + | 356553958 |
|  | G19833-8-016 | 59089215..59089961 | + | 356552036 |
|  | G19833-8-017 | 59097170..59097610 | - | None |
|  | G19833-8-018 | 59100341..59105023 | + | 356551832 |
|  | G19833-8-019 | 59106028..59109778 | - | 356551757 |
|  | G19833-8-020 | 59111538..59113298 | + | 356551676 |
|  | G19833-8-021 | 59114313..59117318 | + | 356551711 |
|  | G19833-8-022 | 59119336..59123184 | + | 356551632 |
|  | G19833-8-023 | 59129339..59133917 | + | 357495375 |
|  | G19833-8-024 | 59135480..59138337 | - | 357495381 |
|  | G19833-8-025 | 59138530..59143500 | - | 356499489 |
|  | G19833-8-026 | 59143887..59151976 | + | 402745687 |
|  | G19833-8-027 | 59153777..59156699 | + | 356551512 |
|  | G19833-8-028 | 59157046..59162193 | - | 363807774 |
|  | G19833-8-029 | 59162654..59169038 | + | 356551432 |
|  | G19833-8-030 | 59169747..59171662 | - | 351723325 |
|  | G19833-8-031 | 59175991..59177626 | - | 351723325 |
|  | G19833-8-032 | 59183838..59186640 | - | 351726171 |
|  | G19833-8-033 | 59187497..59188834 | - | 351726284 |
|  | G19833-8-034 | 59191985..59194729 | + | 356553454 |
|  | G19833-8-035 | 59198597..59201718 | + | 357495457 |
|  | G19833-8-036 | 59204807..59210681 | + | 356554593 |
|  | G19833-8-037 | 59211437..59216139 | - | 356499495 |
|  | G19833-8-038 | 59222877..59225525 | + | 356554509 |
|  | G19833-8-039 | 59226615..59227694 | - | None |
|  | G19833-8-040 | 59228273..59229036 | - | 356554488 |
|  | G19833-8-041 | 59230006..59231273 | - | 356501739 |
|  | G19833-8-042 | 59234393..59238795 | - | 356554441 |
|  | G19833-8-043 | 59241985..59248695 | - | 356553450 |
|  | G19833-8-044 | 59250203..59254296 | + | 356559534 |
|  | G19833-8-045 | 59254651..59258961 | + | 356554340 |
|  | G19833-8-046 | 59259635..59261423 | - | 356554296 |
|  | G19833-8-047 | 59270287..59271992 | + | 356499499 |
|  | G19833-8-048 | 59272338..59273270 | - | 356501745 |
|  | G19833-8-049 | 59273676..59278360 | + | 356553444 |
|  | G19833-8-050 | 59278666..59279652 | - | 356553442 |
|  | G19833-8-051 | 59282482..59285813 | - | 270340065 |
|  | G19833-8-052 | 59288947..59292470 | - | 356499501 |
|  | G19833-8-053 | 59300431..59308736 | - | 356553440 |
|  | G19833-8-054 | 59310300..59313541 | + | 129586 |
|  | G19833-8-055 | 59314171..59316771 | - | 356501751 |
|  | G19833-8-056 | 59318374..59321129 | - | 356501755 |
|  | G19833-8-057 | 59322023..59325776 | - | 357495591 |
|  | G19833-8-058 | 59327180..59330998 | + | 356553641 |
|  | G19833-8-059 | 59331107..59333021 | - | 356553438 |
|  | G19833-8-060 | 59333242..59335980 | + | None |
|  | G19833-8-061 | 59337672..59339680 | - | 396582354 |
|  | G19833-8-062 | 59341579..59343832 | - | 396582356 |
|  | G19833-8-063 | 59344106..59347127 | - | 396582357 |
|  | G19833-8-064 | 59347945..59351756 | - | 356553731 |
|  | G19833-8-065 | 59352092..59359545 | + | 396582359 |
|  | G19833-8-066 | 59361139..59361706 | - | 396582360 |
|  | G19833-8-067 | 59364819..59370224 | + | 396582347 |
|  | G19833-8-068 | 59371533..59373071 | - | 396582346 |
|  | G19833-8-069 | 59373530..59376702 | - | 396582348 |
|  | G19833-8-070 | 59380960..59383790 | - | 396582349 |
|  | G19833-8-071 | 59385163..59389458 | - | 396582349 |
|  | G19833-8-072 | 59391490..59396901 | - | 356499513 |
|  | G19833-8-073 | 59402438..59407664 | - | 356553431 |
|  | G19833-8-074 | 59410969..59417500 | + | 356553429 |
|  | G19833-8-075 | 59417514..59419328 | - | 356552735 |
|  | G19833-8-076 | 59419376..59421343 | + | 351725127 |
|  | G19833-8-077 | 59422378..59424222 | - | 356499523 |
|  | G19833-8-078 | 59425249..59426212 | - | 218937660 |
|  | G19833-8-079 | 59428360..59431059 | + | 356499521 |
|  | G19833-8-080 | 59432079..59450802 | - | 356553425 |
|  | G19833-8-081 | 59450902..59451535 | + | None |
|  | G19833-8-082 | 59452395..59454934 | - | 356501763 |
|  | G19833-8-083 | 59456460..59459921 | - | 351726644 |
|  | G19833-8-084 | 59461689..59471915 | + | 356553423 |
|  | G19833-8-085 | 59472431..59477927 | + | 356553421 |
|  | G19833-8-086 | 59478116..59480513 | + | 359807161 |
|  | G19833-8-087 | 59482562..59487292 | - | 356499527 |
|  | G19833-8-088 | 59487706..59493357 | + | 356551652 |
|  | G19833-8-089 | 59493460..59494605 | + | 356551651 |
|  | G19833-8-090 | 59496915..59499450 | + | 270342091 |
|  | G19833-8-091 | 59499459..59503631 | - | 218937662 |
|  | G19833-8-092 | 59503751..59507391 | - | 218937660 |
|  | G19833-8-093 | 59598792..59600484 | + | None |
|  | G19833-8-094 | 59602380..59603372 | - | 351726838 |
|  | G19833-8-095 | 59605198..59613444 | + | 356501771 |
|  | G19833-8-096 | 59615222..59620875 | + | 356554044 |
|  | G19833-8-097 | 59622708..59628466 | - | 356553419 |
|  | G19833-8-098 | 59629339..59636865 | - | 356501775 |
|  | G19833-8-099 | 59637238..59643108 | - | 356552456 |
|  | G19833-8-100 | 59643250..59643853 | - | None |
|  | G19833-8-101 | 59644202..59645982 | - | 356501775 |
|  | G19833-8-102 | 59647554..59650332 | + | 356501777 |
|  | G19833-8-103 | 59650529..59651224 | - | None |
|  | G19833-8-104 | 59651957..59652411 | + | 356497526 |
|  | G19833-8-105 | 59652628..59660407 | + | 218937660 |
|  | 231733-8-001 | 1931..3177 | + | 357495321 |
|  | 231733-8-002 | 4404..10080 | - | 357493209 |
|  | 231733-8-003 | 10135..11344 | - | 357493209 |
|  | 231733-8-004 | 13860..22394 | - | 357493209 |
|  | 231733-8-005 | 24691..31932 | - | 357460465 |
|  | 231733-8-006 | 32042..33238 | - | 357493209 |
|  | 231733-8-007 | 34234..34991 | - | None |
|  | 231733-8-008 | 35740..42245 | + | 356501703 |
|  | 231733-8-009 | 44524..46557 | - | 356552149 |
|  | 231733-8-010 | 46759..47309 | - | 356552149 |
|  | 231733-8-011 | 48834..50966 | + | 229368631 |
|  | 231733-8-012 | 51001..51761 | - | 357495337 |
|  | 231733-8-013 | 58461..69877 | + | 356553458 |
|  | 231733-8-014 | 70416..71166 | + | 356552037 |
|  | 231733-8-015 | 78565..79354 | - | None |
|  | 231733-8-016 | 82032..86709 | - | 356551832 |
|  | 231733-8-017 | 87714..91470 | - | 356551757 |
|  | 231733-8-018 | 93206..94966 | + | 356551676 |
|  | 231733-8-019 | 95828..98762 | + | 356551711 |
|  | 231733-8-020 | 100898..104736 | + | 356551632 |
|  | 231733-8-021 | 109568..115078 | + | 357495357 |
|  | 232029-8-001 | 1388..4450 | + | 356551512 |
|  | 232029-8-002 | 4801..9866 | - | 363807774 |
|  | 232029-8-003 | 10754..17141 | + | 356551432 |
|  | 232029-8-004 | 17858..20060 | - | 351723325 |
|  | 232029-8-005 | 23839..25842 | - | 351723325 |
|  | 232029-8-006 | 33102..36113 | - | 351726171 |
|  | 232029-8-007 | 36992..38340 | - | 62710746 |
|  | 232029-8-008 | 41493..44177 | + | 356553454 |
|  | 232029-8-009 | 47664..50898 | + | 357495457 |
|  | 232029-8-010 | 54016..59890 | + | 356554593 |
|  | 232029-8-011 | 60667..65508 | - | 356499495 |
|  | 232029-8-012 | 72108..74945 | + | 356554509 |
|  | 232029-8-013 | 75084..77130 | - | 356501733 |
|  | 232029-8-014 | 77708..78474 | - | 312050704 |
|  | 232029-8-015 | 79443..82222 | - | 356501739 |
|  | 232029-8-016 | 83937..88333 | - | 356554441 |
|  | 232029-8-017 | 91187..91947 | + | 359806707 |
|  | 232029-8-018 | 92213..98397 | - | 356553450 |
|  | 232029-8-019 | 101432..103972 | + | 356559534 |
|  | 232029-8-020 | 104327..107124 | + | 356554340 |
|  | 232029-8-021 | 109508..111304 | - | 356554296 |
|  | 232029-8-022 | 120496..122161 | + | 356499499 |
|  | 232029-8-023 | 122868..123456 | - | 356501745 |
|  | 232029-8-024 | 124229..128829 | + | 356553444 |
|  | 232029-8-025 | 129000..129886 | - | 356553442 |
|  | 232701-8-001 | 3562..10186 | - | 356551652 |
|  | 232701-8-002 | 10351..15218 | + | 356499527 |
|  | 232701-8-003 | 17089..19212 | - | 359807161 |
|  | 232701-8-004 | 19338..23560 | - | 356553421 |
|  | 232701-8-005 | 25113..34594 | - | 356553423 |
|  | 232701-8-006 | 36865..40493 | + | 351726044 |
|  | 232701-8-007 | 42491..59132 | + | 356553425 |
|  | 232701-8-008 | 62281..69187 | + | 356553425 |
|  | 232701-8-009 | 70155..72872 | - | 356499521 |
|  | 232701-8-010 | 78072..78750 | + | 356499523 |
|  | 232701-8-011 | 79570..81782 | - | 351725127 |
|  | 232701-8-012 | 81828..83719 | + | 356252735 |
|  | 232701-8-013 | 84091..92312 | - | 356553429 |
|  | 232701-8-014 | 93887..100476 | + | 356553431 |
|  | 232701-8-015 | 104516..110372 | + | 356553425 |
|  | 232701-8-016 | 113984..114536 | + | 396582350 |
|  | 232701-8-017 | 114632..117357 | + | 396582349 |
|  | 232701-8-018 | 119058..121313 | + | 396582348 |
|  | 232701-8-019 | 127947..128089 | + | 396582346 |
|  | 232701-8-020 | 128102..130242 | - | 396582345 |
|  | 232701-8-021 | 132737..133012 | + | 396582360 |
|  | 232701-8-022 | 133283..136852 | - | 396582366 |
|  | 232701-8-023 | 139286..140575 | + | 396582359 |
|  | 232701-8-024 | 140997..146280 | + | 396582358 |
|  | 232701-8-025 | 146636..150662 | + | 396582357 |
|  | 232701-8-026 | 150936..153818 | + | 396582346 |
|  | 232701-8-027 | 154254..156552 | + | 396582355 |
|  | 232701-8-028 | 157112..160208 | - | 356499507 |
|  | 232701-8-029 | 160321..162237 | + | 356553438 |
|  | 232701-8-030 | 162439..166678 | - | 356553641 |
|  | 232701-8-031 | 167796..172096 | + | 351726072 |
|  | 232701-8-032 | 172808..175269 | + | 356501755 |
|  | 232701-8-033 | 176754..179425 | + | 356501751 |
|  | 232701-8-034 | 180032..183149 | - | 129586 |
|  | 232701-8-035 | 185437..192973 | + | 356553440 |
|  | 231171-8-001 | 2535..5496 | + | 218437660 |
|  | 231171-8-002 | 9776..12987 | - | 260753266 |
|  | 231171-8-003 | 13119..25074 | - | 267753266 |
|  | 231171-8-004 | 25436..26688 | - | 270342091 |
|  | 231171-8-005 | 30962..33200 | + | 260753266 |
|  | 231171-8-006 | 37134..39836 | + | None |
|  | 231171-8-007 | 39950..42385 | - | 356501776 |
|  | 231171-8-008 | 43859..45855 | + | 356501775 |
|  | 231171-8-009 | 46550..52433 | + | 356501773 |
|  | 231171-8-010 | 52626..60355 | + | 356501773 |
|  | 231171-8-011 | 61175..67070 | + | 356553419 |
|  | 231171-8-012 | 69024..74417 | - | 356554044 |
|  | 231171-8-013 | 76773..83574 | - | 356501771 |
|  | 231171-8-014 | 85751..86773 | + | 351726838 |
|  | 231171-8-015 | 89780..90904 | - | 218937662 |
| 10 | G19833-10-001 | 39949354..39949354 | + | 308791059 |
|  | G19833-10-002 | 39958785..39958785 | + | 356520085 |
|  | G19833-10-003 | 39964418..39964418 | - | 356559157 |
|  | G19833-10-004 | 39973500..39973500 | + | 356559157 |
|  | G19833-10-005 | 39974706..39974706 | - | 356559136 |
|  | G19833-10-006 | 39982763..39982763 | + | 255638476 |
|  | G19833-10-007 | 40001521..40001521 | - | 351723909 |
|  | G19833-10-008 | 40013558..40013558 | + | 350560177 |
|  | G19833-10-009 | 40017422..40017422 | + | 356535583 |
|  | G19833-10-010 | 40021920..40021920 | - | 350560181 |
|  | G19833-10-011 | 40025592..40025592 | - | 356538735 |
|  | G19833-10-012 | 40031999..40031999 | - | 388504064 |
|  | G19833-10-013 | 40034002..40034002 | - | 359807187 |
|  | G19833-10-014 | 40040225..40040225 | - | 356522422 |
|  | G19833-10-015 | 40050362..40050362 | - | 356560185 |
|  | G19833-10-016 | 40052034..40052034 | + | 16343113 |
|  | G19833-10-017 | 40059428..40059428 | - | 356522426 |
|  | G19833-10-018 | 40067781..40067781 | - | 356579990 |
|  | G19833-10-019 | 40073426..40073426 | + | 356522428 |
|  | G19833-10-020 | 40074600..40074600 | - | 356520011 |
|  | G19833-10-021 | 40082889..40082889 | - | 357514173 |
|  | G19833-10-022 | 40084942..40084942 | - | None |
|  | G19833-10-023 | 40092969..40092969 | - | 356551116 |
|  | G19833-10-024 | 40097696..40097696 | + | 350520051 |
|  | G19833-10-025 | 40099656..40099656 | + | 357514181 |
|  | G19833-10-026 | 40102970..40102970 | + | 207558416 |
|  | G19833-10-027 | 40107869..40107869 | - | 356519905 |
|  | G19833-10-028 | 40111357..40111357 | - | 356519905 |
|  | G19833-10-029 | 40124234..40124234 | - | 356519905 |
|  | G19833-10-030 | 40137428..40137428 | - | 356519889 |
|  | G19833-10-031 | 40147263..40147263 | - | 358249042 |
|  | G19833-10-032 | 40163771..40163771 | + | 356570175 |
|  | G19833-10-033 | 40171819..40171819 | + | 356522412 |
|  | G19833-10-034 | 40177418..40177418 | - | 356558995 |
|  | G19833-10-035 | 40181372..40181372 | - | 225442069 |
|  | G19833-10-036 | 40186174..40186174 | - | 351721438 |
|  | G19833-10-037 | 40191469..40191469 | - | 356522410 |
|  | G19833-10-038 | 40204206..40204206 | - | 356522408 |
|  | G19833-10-039 | 40213525..40213525 | - | 356519838 |
|  | G19833-10-040 | 40234658..40234658 | + | 356558969 |
|  | G19833-10-041 | 40250988..40250988 | + | 356519826 |
|  | G19833-10-042 | 40259112..40259112 | + | 356558954 |
|  | G19833-10-043 | 40261376..40261376 | - | 320117871 |
|  | G19833-10-044 | 40268321..40268321 | + | 38194929 |
|  | G19833-10-045 | 40271885..40271885 | - | None |
|  | G19833-10-046 | 40275761..40275761 | - | None |
|  | G19833-10-047 | 40278502..40278502 | + | 357514047 |
|  | G19833-10-048 | 40285078..40285078 | - | 356560845 |
|  | G19833-10-049 | 40303538..40303538 | + | 356519798 |
|  | G19833-10-050 | 40306670..40306670 | + | 356575040 |
|  | G19833-10-051 | 40308113..40308113 | - | 147776299 |
|  | G19833-10-052 | 40312438..40312438 | + | 312059257 |
|  | G19833-10-053 | 40318928..40318928 | + | 356548932 |
|  | G19833-10-054 | 40332132..40332132 | - | 350519790 |
|  | G19833-10-055 | 40346909..40346909 | - | None |
|  | G19833-10-056 | 40376847..40376847 | + | 37196706 |
|  | G19833-10-057 | 40380559..40380559 | - | 388497288 |
|  | G19833-10-058 | 40387369..40387369 | + | 356567748 |
|  | G19833-10-059 | 40391257..40391257 | - | 356522404 |
|  | G19833-10-060 | 40396500..40396500 | + | 356519778 |
|  | G19833-10-061 | 40403064..40403064 | - | 356558880 |
|  | G19833-10-062 | 40411647..40411647 | - | 7242813 |
|  | G19833-10-063 | 40417333..40417333 | - | 356519733 |
|  | G19833-10-064 | 40422418..40422418 | - | 196122020 |
|  | G19833-10-065 | 40444507..40444507 | + | 356522402 |
|  | G19833-10-066 | 40452302..40452302 | - | 62715391 |
|  | G19833-10-067 | 40469549..40469549 | - | 356560162 |
|  | G19833-10-068 | 40476093..40476093 | - | 225442140 |
|  | G19833-10-069 | 40489221..40489221 | + | 260753266 |
|  | G19833-10-070 | 40492414..40492414 | + | None |
|  | G19833-10-071 | 40493817..40493817 | + | None |
|  | G19833-10-072 | 40500183..40500183 | - | 190529025 |
|  | G19833-10-073 | 40502110..40502110 | - | 356522398 |
|  | G19833-10-074 | 40518778..40518778 | - | 356560158 |
|  | G19833-10-075 | 40530505..40530505 | - | 357513949 |
|  | G19833-10-076 | 40556507..40556507 | + | 356522392 |
|  | G19833-10-077 | 40564042..40564042 | + | 357513901 |
|  | G19833-10-078 | 40573346..40573346 | + | 22094359 |
|  | G19833-10-079 | 40574836..40574836 | + | 109391001 |
|  | G19833-10-080 | 40576480..40576480 | + | 259191134 |
|  | G19833-10-081 | 40577838..40577838 | - | 308784976 |
|  | G19833-10-082 | 40581115..40581115 | + | 225453228 |
|  | G19833-10-083 | 40583701..40583701 | - | 356558721 |
|  | G19833-10-084 | 40588731..40588731 | + | 9759493 |
|  | G19833-10-085 | 40591957..40591957 | + | 356522390 |
|  | G19833-10-086 | 40600525..40600525 | - | 356558749 |
|  | G19833-10-087 | 40609146..40609146 | - | 357513925 |
|  | G19833-10-088 | 40614557..40614557 | - | 312062530 |
|  | G19833-10-089 | 40617293..40617293 | + | 123359649 |
|  | G19833-10-090 | 40620114..40620114 | + | 9989054 |
|  | G19833-10-091 | 40624576..40624576 | + | 356558721 |
|  | G19833-10-092 | 40636476..40636476 | + | 356558721 |
|  | G19833-10-093 | 40642555..40642555 | + | 356558721 |
|  | G19833-10-094 | 40645206..40645206 | - | 357470935 |
|  | G19833-10-095 | 40650265..40650265 | + | 315937172 |
|  | G19833-10-096 | 40659021..40659021 | + | 356522396 |
|  | G19833-10-097 | 40669283..40669283 | + | 356522390 |
|  | G19833-10-098 | 40675793..40675793 | + | 356558721 |
|  | G19833-10-099 | 40681157..40681157 | - | 356522390 |
|  | G19833-10-100 | 40686071..40686071 | - | 356558721 |
|  | G19833-10-101 | 40709755..40709755 | + | 356522390 |
|  | G19833-10-102 | 40711009..40711009 | + | None |
|  | G19833-10-103 | 40717274..40718231 | + | 255560661 |
|  | G19833-10-104 | 40718377..40721735 | - | 255546017 |
|  | G19833-10-105 | 40742791..40746146 | - | 356561747 |
|  | G19833-10-106 | 40758023..40767138 | - | 356519582 |
|  | G19833-10-107 | 40767465..40770735 | + | 356539804 |
|  | G19833-10-108 | 40772303..40775828 | - | 356519574 |
|  | G19833-10-109 | 40777812..40780179 | + | 312042445 |
|  | G19833-10-110 | 40790906..40797322 | + | 356519562 |
|  | G19833-10-111 | 40800976..40803154 | - | None |
|  | G19833-10-112 | 40803451..40809202 | + | 356561291 |
|  | G19833-10-113 | 40822496..40824271 | - | 356522390 |
|  | G19833-10-114 | 40824379..40826463 | + | 171611480 |
|  | G19833-10-115 | 40831741..40836200 | - | None |
|  | G19833-10-116 | 40840180..40841946 | - | 356522390 |
|  | G19833-10-117 | 40845143..40852404 | + | 357472507 |
|  | G19833-10-118 | 40945027..40948555 | + | 124484391 |
|  | G19833-10-119 | 40954557..40957737 | + | 315727963 |
|  | G19833-10-120 | 40962596..40967207 | + | 351727220 |
|  | G19833-10-121 | 40967685..40971415 | + | 356561047 |
|  | G19833-10-122 | 40974570..40975917 | - | 356519521 |
|  | G19833-10-123 | 40976101..40976771 | + | 162291207 |
|  | G19833-10-124 | 40978466..40980093 | + | 312049928 |
|  | G19833-10-125 | 40982559..40983915 | - | 356560143 |
|  | G19833-10-126 | 40988361..40990890 | - | 255640830 |
|  | G19833-10-127 | 40993611..40995601 | - | 255640830 |
|  | G19833-10-128 | 41004994..41006052 | - | 356560143 |
|  | G19833-10-129 | 41007578..41007927 | - | 312057571 |
|  | G19833-10-130 | 41019085..41038027 | - | 357513851 |
|  | 233269-10-001 | 6..2080 | - | 353685479 |
|  | 233269-10-002 | 6232..7637 | + | 357514359 |
|  | 233269-10-003 | 13586..15460 | + | 356560202 |
|  | 233269-10-004 | 16127..18952 | - | 356559270 |
|  | 233269-10-005 | 25505..28849 | - | 356520176 |
|  | 233269-10-006 | 31513..32168 | - | None |
|  | 233269-10-007 | 33965..35373 | + | 187432434 |
|  | 233269-10-008 | 36376..50171 | + | 356522450 |
|  | 233269-10-009 | 59411..61597 | + | 356520174 |
|  | 233269-10-010 | 61744..64191 | + | 356560200 |
|  | 233269-10-011 | 67612..70819 | + | 6093739 |
|  | 233269-10-012 | 76224..82925 | + | 356520168 |
|  | 233269-10-013 | 84434..89684 | - | 255441987 |
|  | 233269-10-014 | 94674..97122 | + | 351727044 |
|  | 233269-10-015 | 99294..108380 | - | 356522446 |
|  | 233269-10-016 | 114961..116697 | + | 356559212 |
|  | 233269-10-017 | 118052..121038 | + | 357574303 |
|  | 233269-10-018 | 125700..126214 | - | 357208876 |
|  | 233269-10-019 | 129157..137717 | - | 357514291 |
|  | 233269-10-020 | 137819..139322 | - | 225016149 |
|  | 233269-10-021 | 143934..145162 | + | 359806545 |
|  | 233269-10-022 | 145595..147825 | + | None |
|  | 233269-10-023 | 152366..159043 | + | 356522438 |
|  | 233269-10-024 | 161900..164435 | + | 356522438 |
|  | 233269-10-025 | 170796..174747 | + | 356522438 |
|  | 233269-10-026 | 177991..179334 | + | 356560196 |
|  | 233269-10-027 | 183838..187231 | + | 356559169 |
|  | 233269-10-028 | 187344..191840 | - | 356522439 |
|  | 233269-10-029 | 192343..194453 | - | 357467641 |
|  | 233269-10-030 | 199095..200916 | - | 353685490 |
|  | 233269-10-031 | 214389..217056 | + | 351725369 |
|  | 233269-10-032 | 227038..228865 | + | 4753889 |
|  | 233269-10-033 | 229052..238547 | + | 356520157 |
|  | 233269-10-034 | 239434..243534 | - | 356563522 |
|  | 233269-10-035 | 256570..267448 | + | 356520085 |
|  | 233269-10-036 | 270677..273095 | - | 356559157 |
|  | 233269-10-037 | 274278..281856 | + | 356559147 |
|  | 233269-10-038 | 281998..283065 | - | 356559136 |
|  | 233269-10-039 | 289206..291495 | + | 255638476 |
|  | 233269-10-040 | 306360..310745 | - | 357479933 |
|  | 233269-10-041 | 315076..322614 | + | 357514119 |
|  | 233269-10-042 | 323580..326007 | + | 356535583 |
|  | 233269-10-043 | 326348..331496 | - | 356560181 |
|  | 233269-10-044 | 333323..336193 | - | 356538735 |
|  | 233269-10-045 | 338697..341086 | - | 356559059 |
|  | 233269-10-046 | 341729..342871 | - | 356807187 |
|  | 233269-10-047 | 347126..349980 | - | 356522422 |
|  | 233269-10-048 | 350948..360667 | - | 356560185 |
|  | 233269-10-049 | 360989..362343 | + | 16343113 |
|  | 233269-10-050 | 362422..369714 | - | 357414165 |
|  | 233269-10-051 | 375790..378090 | - | 356519990 |
|  | 233269-10-052 | 382368..383636 | + | 356522428 |
|  | 233269-10-053 | 384382..384820 | - | 356520011 |
|  | 233269-10-054 | 388927..393138 | - | 357514176 |
|  | 233269-10-055 | 398871..402865 | - | 356559116 |
|  | 233269-10-056 | 403893..407289 | + | 356520051 |
|  | 233269-10-057 | 408173..409327 | + | 357514181 |
|  | 233269-10-058 | 412243..412637 | + | 207558416 |
|  | 233269-10-059 | 412949..417536 | - | 356519905 |
|  | 233269-10-060 | 417581..421024 | - | 356519905 |
|  | 233269-10-061 | 422268..433919 | - | 356519905 |
|  | 233269-10-062 | 436808..446970 | - | 356519889 |
|  | 233269-10-063 | 453387..456852 | - | 358249042 |
|  | 233269-10-064 | 469992..473493 | + | 356560175 |
|  | 232627-10-001 | 477..3005 | - | None |
|  | 232627-10-002 | 3376..4251 | + | 312055677 |
|  | 232627-10-003 | 4608..6393 | + | None |
|  | 232627-10-004 | 6794..11252 | + | 317106737 |
|  | 232627-10-005 | 11269..12376 | + | 171501994 |
|  | 232627-10-006 | 14451..15095 | - | 27397628 |
|  | 232627-10-007 | 15356..17502 | + | 351560171 |
|  | 232627-10-008 | 17887..19809 | + | 87162935 |
|  | 232627-10-009 | 20829..23487 | - | 356522410 |
|  | 232627-10-010 | 31859..45993 | - | 255583954 |
|  | 232627-10-011 | 61524..67270 | + | 18400268 |
|  | 232627-10-012 | 80104..83011 | + | 356519826 |
|  | 232627-10-013 | 87690..90649 | + | 356558954 |
|  | 232627-10-014 | 96624..100004 | + | 320117871 |
|  | 232627-10-015 | 101508..102674 | - | 38194929 |
|  | 232627-10-016 | 102766..104102 | - | None |
|  | 232627-10-017 | 107194..108608 | + | 357514047 |
|  | 232627-10-018 | 109610..114230 | - | 260753266 |
|  | 232627-10-019 | 119236..122479 | - | 356560845 |
|  | 232627-10-020 | 135993..141635 | + | 356519798 |
|  | 232627-10-021 | 149640..149815 | - | 312059257 |
|  | 232627-10-022 | 153231..156112 | + | 356558932 |
|  | 232627-10-023 | 166442..168574 | - | 356519780 |
|  | 232627-10-024 | 176510..177238 | - | None |
|  | 232393-10-001 | 7706..11908 | + | 37196706 |
|  | 232393-10-002 | 15358..15968 | - | 312040269 |
|  | 232393-10-003 | 16881..22763 | + | 356567748 |
|  | 232393-10-004 | 23008..26270 | - | 356522404 |
|  | 232393-10-005 | 27642..32550 | + | 356519778 |
|  | 232393-10-006 | 38289..39089 | - | 356558880 |
|  | 232393-10-007 | 46413..47121 | - | 7242813 |
|  | 232393-10-008 | 51765..52773 | - | 356519733 |
|  | 232393-10-009 | 56873..57855 | - | 196122020 |
|  | 232393-10-010 | 72800..74765 | + | 356522402 |
|  | 232393-10-011 | 81023..82574 | - | 62715391 |
|  | 232393-10-012 | 95247..99305 | - | 356560162 |
|  | 232393-10-013 | 100461..105912 | - | 351722369 |
|  | 232393-10-014 | 106780..109444 | + | 312055784 |
|  | 232393-10-015 | 111944..117624 | + | 260753266 |
|  | 232393-10-016 | 118287..119145 | + | None |
|  | 232393-10-017 | 122166..123591 | - | 356522398 |
|  | 232393-10-018 | 136240..140553 | - | 356560158 |
|  | 232393-10-019 | 143282..151513 | - | 356519645 |
|  | 232393-10-020 | 156523..159933 | - | None |
|  | 225305-10-001 | 9496..17936 | + | 356522392 |
|  | 227881-10-001 | 2002..3016 | - | 356558721 |
|  | 227881-10-002 | 11453..13020 | + | 356522390 |
|  | 227881-10-003 | 16149..16871 | - | None |
|  | 227881-10-004 | 20218..25870 | - | 356558749 |
|  | 227881-10-005 | 25992..36174 | + | 357513925 |
|  | 233141-10-001 | 1644..2885 | + | None |
|  | 233141-10-002 | 6415..11763 | - | 51854301 |
|  | 233141-10-003 | 13594..20654 | - | 353685479 |
|  | 233141-10-004 | 24619..25453 | - | 356522390 |
|  | 233141-10-005 | 25551..28026 | + | 124360558 |
|  | 233141-10-006 | 31169..33542 | + | 356558721 |
|  | 233141-10-007 | 37228..39059 | + | 356558721 |
|  | 233141-10-008 | 42779..47062 | + | 356558721 |
|  | 233141-10-009 | 47413..54085 | - | 356522390 |
|  | 233141-10-010 | 57475..63421 | + | 356558721 |
|  | 233141-10-011 | 64258..65884 | + | 356522390 |
|  | 233141-10-012 | 66591..71982 | + | 120529942 |
|  | 233141-10-013 | 73306..74730 | - | 356522390 |
|  | 233141-10-014 | 74786..76925 | - | 356522390 |
|  | 233141-10-015 | 81468..83415 | + | 356548749 |
|  | 233141-10-016 | 86783..87962 | + | 356522390 |
|  | 233141-10-017 | 103020..104051 | + | 304557181 |
|  | 233141-10-018 | 104198..106093 | - | 255546017 |
|  | 233141-10-019 | 130635..133985 | - | 356561747 |
|  | 233141-10-020 | 146383..155485 | - | 356519582 |
|  | 233141-10-021 | 155813..159085 | + | 356539804 |
|  | 233141-10-022 | 160061..164179 | - | 186490872 |
|  | 233141-10-023 | 179633..186058 | + | 356519562 |
|  | 233141-10-024 | 189170..191444 | - | None |
|  | 233141-10-025 | 192089..200542 | + | 124484391 |
|  | 233141-10-026 | 206437..209631 | + | 351727963 |
|  | 233141-10-027 | 214484..219095 | + | 351727220 |
|  | 233141-10-028 | 219574..223305 | + | 356561047 |
|  | 233141-10-029 | 226460..227807 | - | 356519521 |
|  | 233141-10-030 | 227991..228661 | + | 162291207 |
|  | 233141-10-031 | 230314..232034 | + | 312049928 |
|  | 233141-10-032 | 234497..235803 | - | 356560143 |
|  | 233141-10-033 | 240188..242639 | - | 255640830 |
|  | 233141-10-034 | 246548..248053 | - | 312034114 |
|  | 233141-10-035 | 248384..249949 | - | 255640830 |
|  | 233141-10-036 | 256995..257918 | - | 357509013 |
|  | 233141-10-037 | 262474..263377 | - | 356560143 |
|  | 233141-10-038 | 265055..265897 | - | 312057571 |
|  | 233141-10-039 | 273710..312651 | - | 357513851 |
|  | 233141-10-040 | 313195..316187 | - | None |
|  | 233141-10-041 | 316506..321012 | - | 356522380 |
|  | 233141-10-042 | 324559..326384 | + | 351725615 |
|  | 233141-10-043 | 329376..332454 | + | 356522378 |
|  | 233141-10-044 | 338194..340120 | + | 356550408 |
|  | 233141-10-045 | 341035..348148 | - | 356560819 |
|  | 233141-10-046 | 352782..358200 | + | 356525977 |
